# Supplementary material for: Supervised dimensionality reduction for exploration of single-cell data by HSS-LDA
Source: Patterns (N Y). 2022 Jun 24;3(8):100536. doi: 10.1016/j.patter.2022.100536 (PMC9403402; doi:10.1016/j.patter.2022.100536)
Supplement: Document S1. Figures S1–S7 and Table S1 [file mmc1.pdf]

Patterns, Volume *3*

Supplemental information

Supervised dimensionality reduction for exploration of single-cell data by HSS-LDA

Meelad Amouzgar, David R. Glass, Reema Baskar, Inna Averbukh, Samuel C. Kimmey, Albert G. Tsai, Felix J. Hartmann, and Sean C. Bendall

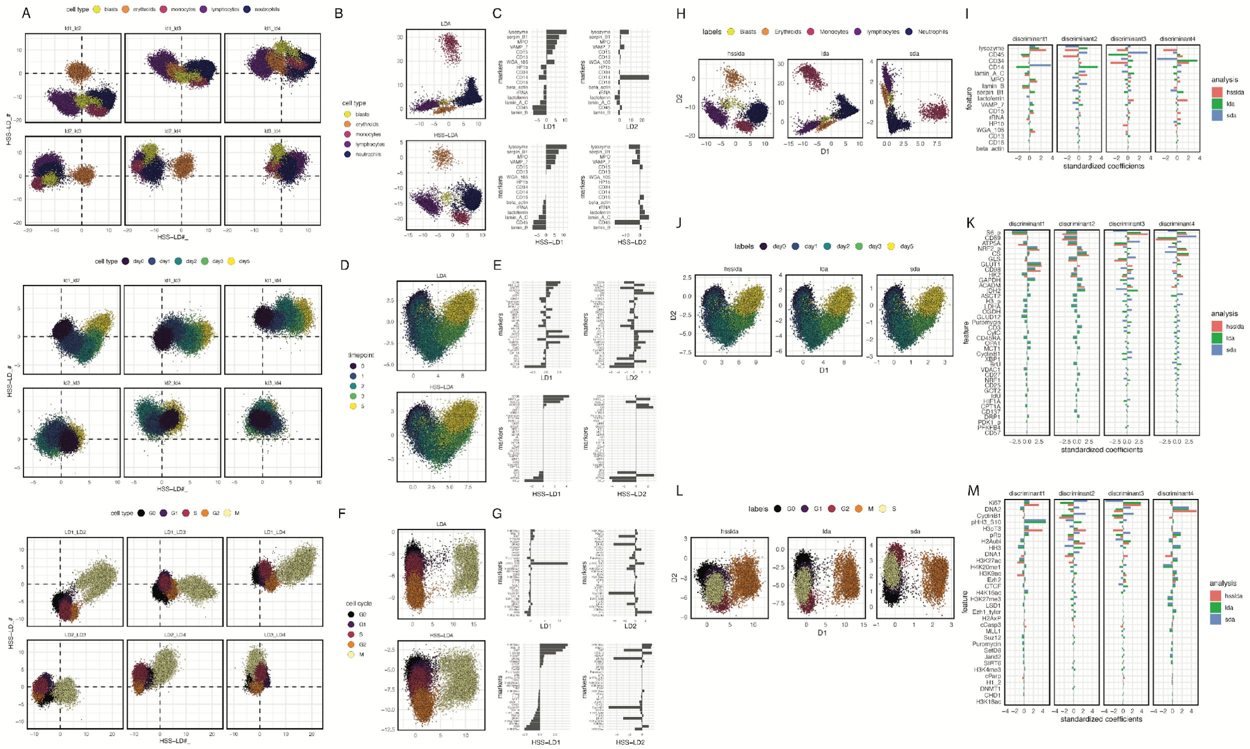

Figure S1: Comparison of LDA to HSS-LDA dimensionality reduction for visualization, feature selection, and feature importance of different mass cytometry datasets.
(A) Pairwise plots of HSS-LDs for the 3 primary mass cytometry datasets. **(B-F)** Results comparing LDA vs HSS-LDA on the same 100,000 subset of cells for each of the 3 primary mass cytometry datasets. **(B, D, F)** LDA and HSS-LDA embeddings colored by cell-type, collection timepoint, and cell cycle phase for the Morphometry, T-cell metabolic regulome, and Chromotyping datasets, respectively. **(C, E, G)** LDA coefficients and HSS-LDA coefficients for the first two linear discriminants sorted by HSS-LDA coefficient values. Features at zero were removed by HSS-LDA. **(H-M)** Comparison of LDA (lda), HSS-LDA (hsslda), and sparseLDA (sda) on the same input cells for each mass cytometry dataset.

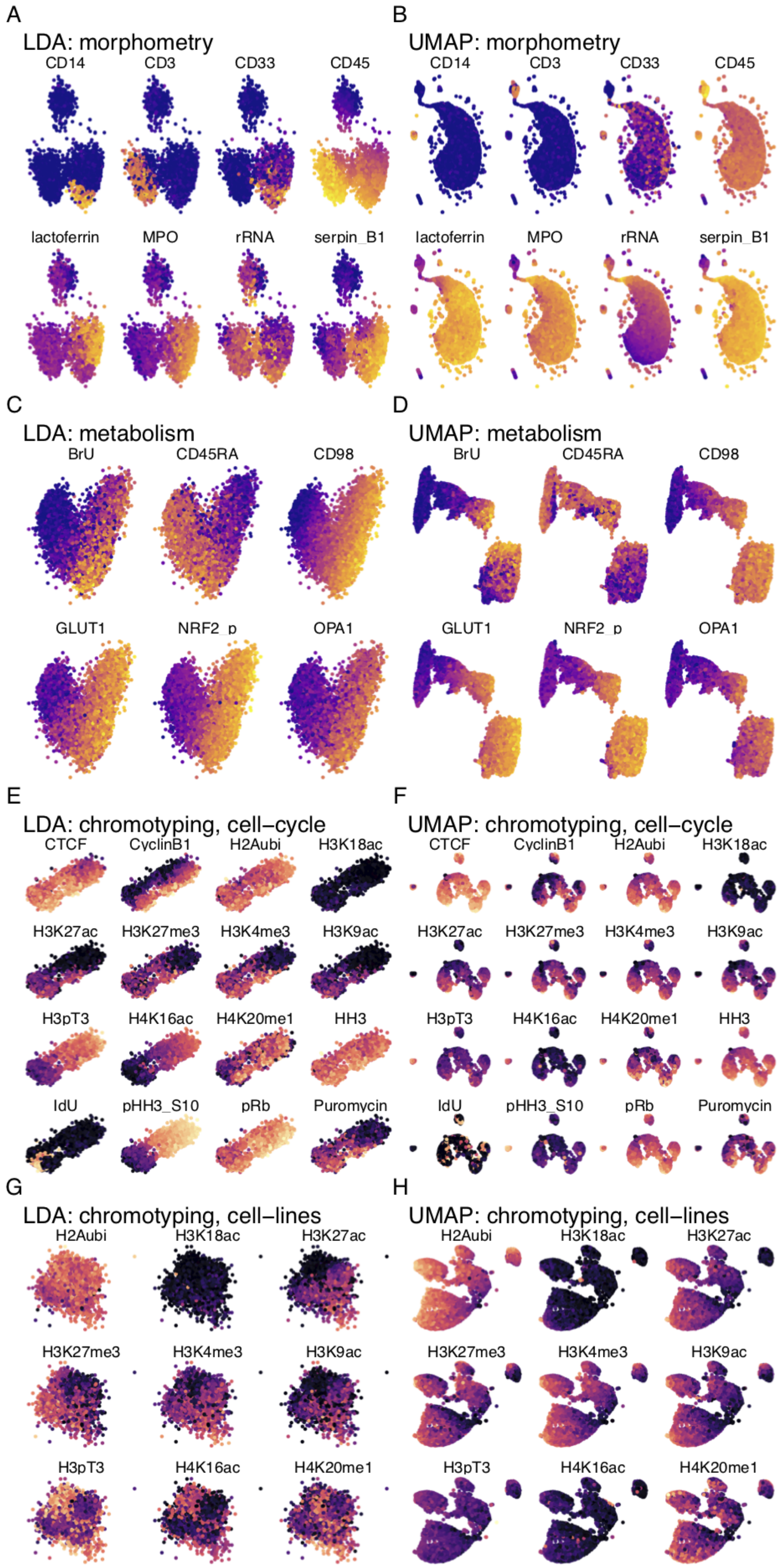
A
LDA: morphometry
CD14
CD3
CD33
CD45
lactoferrin
MPO
rRNA
serpin_B1
B
UMAP: morphometry
CD14
CD3
CD33
CD45
lactoferrin
MPO
rRNA
serpin_B1
C
LDA: metabolism
BrU
CD45RA
CD98
GLUT1
NRF2_p
OPA1
D
UMAP: metabolism
BrU
CD45RA
CD98
GLUT1
NRF2_p
OPA1
E
LDA: chromotyping, cell–cycle
CTCF
CyclinB1
H2Aubi
H3K18ac
H3K27ac
H3K27me3
H3K4me3
H3K9ac
H3pT3
H4K16ac
H4K20me1
HH3
IdU
pHH3_S10
pRb
Puromycin
F
UMAP: chromotyping, cell–cycle
CTCF
CyclinB1
H2Aubi
H3K18ac
H3K27ac
H3K27me3
H3K4me3
H3K9ac
H3pT3
H4K16ac
H4K20me1
HH3
IdU
pHH3_S10
pRb
Puromycin
G
LDA: chromotyping, cell–lines
H2Aubi
H3K18ac
H3K27ac
H3K27me3
H3K4me3
H3K9ac
H3pT3
H4K16ac
H4K20me1
H
UMAP: chromotyping, cell–lines
H2Aubi
H3K18ac
H3K27ac
H3K27me3
H3K4me3
H3K9ac
H3pT3
H4K16ac
H4K20me1

Figure S2: Protein expression of HSS-LDA and UMAP embeddings for different mass cytometry datasets.
(A) Morphometry HSS-LDA embedding colored by protein expression for cell-type labels. **(B)** Morphometry UMAP embedding colored by protein expression for cell-type labels. **(C)** T-cell metabolic regulome HSS-LDA embedding colored by protein expression for for timepoint labels. **(D)** T-cell metabolic regulome UMAP embedding colored by protein expression for timepoint labels. **(E)** Chromotyping HSS-LDA embedding colored by protein expression for cell cycle labels. **(F)** Chromotyping UMAP embedding colored by protein expression for cell cycle labels. **(G)** Chromotyping HSS-LDA embedding colored by protein expression for cell line labels. **(H)** Chromotyping UMAP embedding colored by protein expression for cell line labels.

Supplementary Items
Manuscript_PATTERNS-D-22-00024_v1

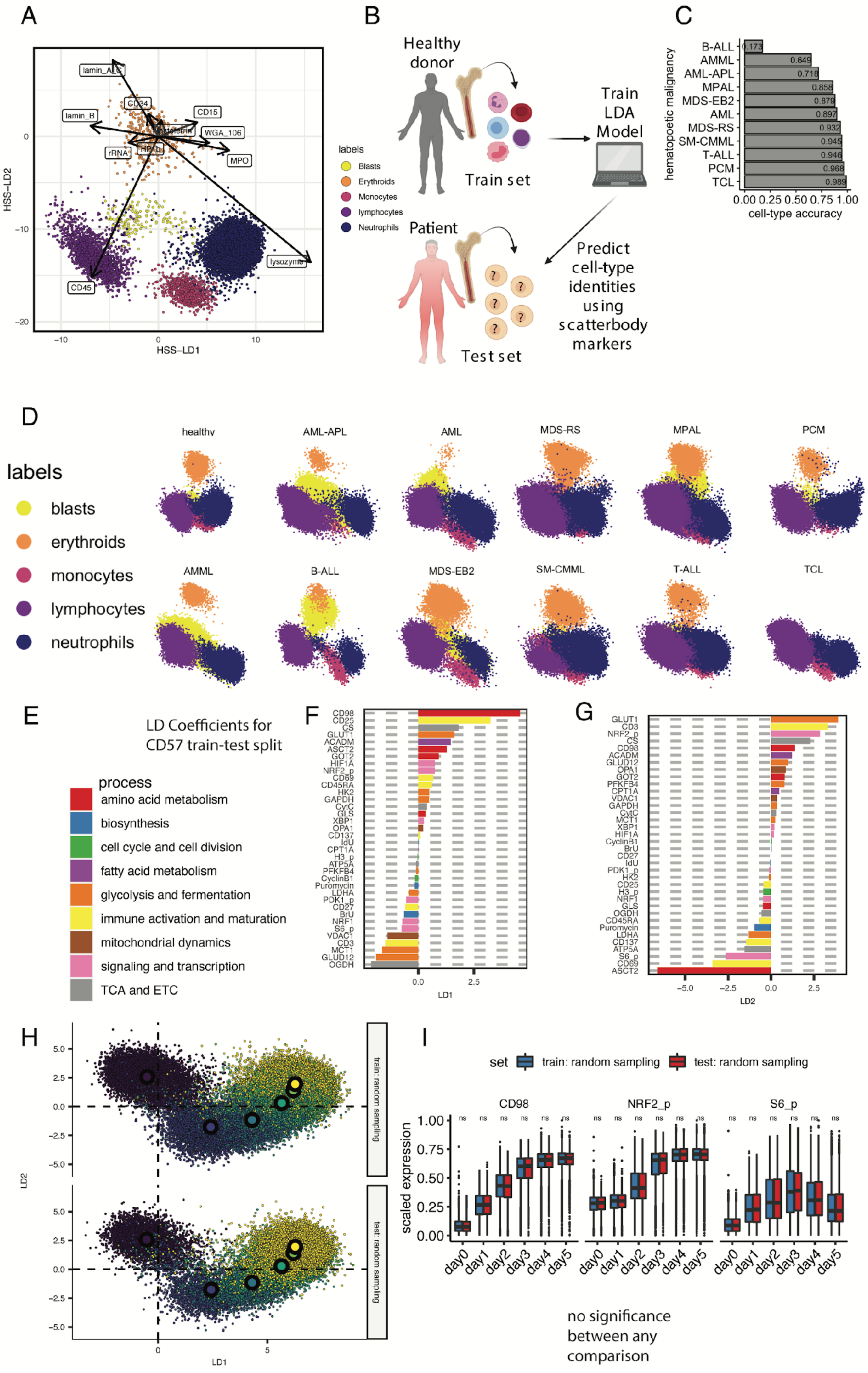

Figure S3: HSS-LDA for predictive analysis of cell-types across different hematopoietic malignancies using scatterbodies, and random sampling of T-cell metabolism dataset for projecting unseen data.
(A) Biaxial plot of LD coefficients representing the magnitude and direction for each feature selected by HSS-LDA. **(B)** Graphical illustration of training HSS-LDA model on immune cells from healthy patients using scatterbody protein markers and tested on immune cells from patients with diverse hematopoietic malignancies for accurate cell-type predictions. **(C)** Accuracy of cell-type predictions across different hematopoietic malignancies using HSS-LDA. **(D)** Biaxial LD embeddings of training set (healthy) and test sets (hematopoietic malignancies) projected onto the healthy LD embedding space. **(E)** Labels for different biological system categories of each marker, derived from Hartmann et al, 2020. **(F-G)** Magnitude and direction of coefficients for all markers in the HSS-LD1 *(E)* and HSS-LD2 *(F)* axes, respectively. **(H)** Biaxial HSS-LD plots of randomly sampled cells independent of CD57 expression and labeled with the centroid point for each timepoint. Supervised learning models are known to have decreased performance on test sets. To test the hypothesis that the metabolically slowed trajectory of $CD57^{high}$ cells seen in *G-I* is not due to decreased performance on the $CD57^{high}$ test set, we randomly sample cells for a train-test split and train the HSS-LDA model. The biaxial HSS-LD plots show there is no change between the training set and test set, indicating the metabolically slowed progression phenotype observed in $CD57^{high}$ cells is not dependent on poor test set performance. **(I)** Boxplot summary of protein expression for randomly sampled cells across each timepoint. Wilcoxon signed-rank test performed between randomly sampled cells across each timepoint shows no significant difference in protein expression between the train or test set, indicating the metabolically slowed phenotype in the $CD57^{high}$ cells versus $CD57^{low}$ (Figure 2) is a true representation of their metabolic trajectory. Wilcoxon signed-rank test performed between $CD57^{low}$ and $CD57^{high}$ cells across each timepoint. *: $p <= 0.05$; **: $p <= 0.01$; ***: $p <= 0.001$; ****: $p <= 0.0001$.

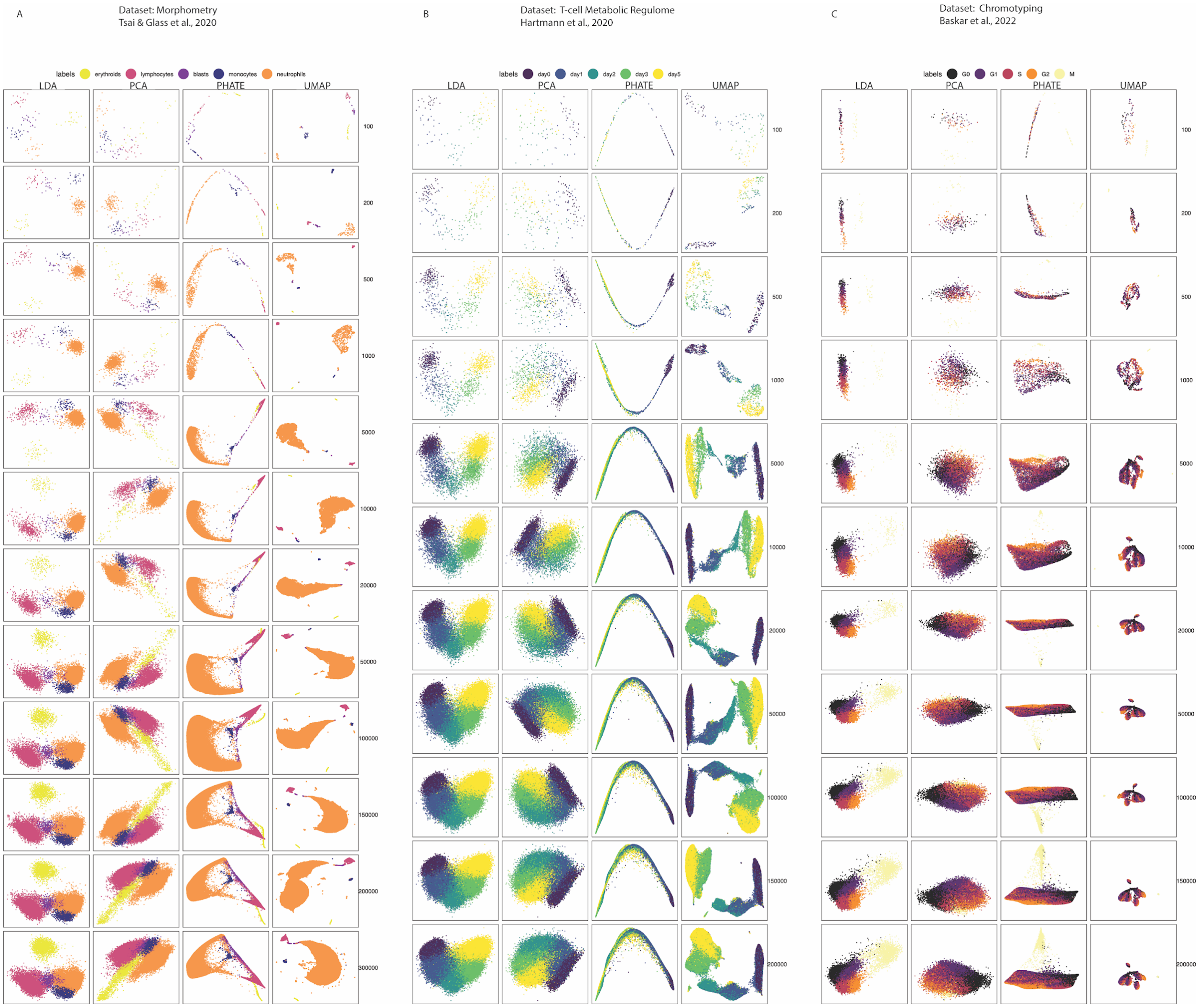

Figure S4: Comparison of biaxial embeddings for LDA, PCA, UMAP, and PHATE across different data subsets.

(A-C) Biaxial visualizations for varying cell-counts across each algorithm using the **(A)** Morphometry for cell-type, **(B)** T-cell Metabolic Regulome for timepoints, and **(C)** Chromotyping for cell cycle datasets. All algorithms benefit from HSS-LDA feature selection, and the feature matrix for each algorithm is the same for each subset of the data.

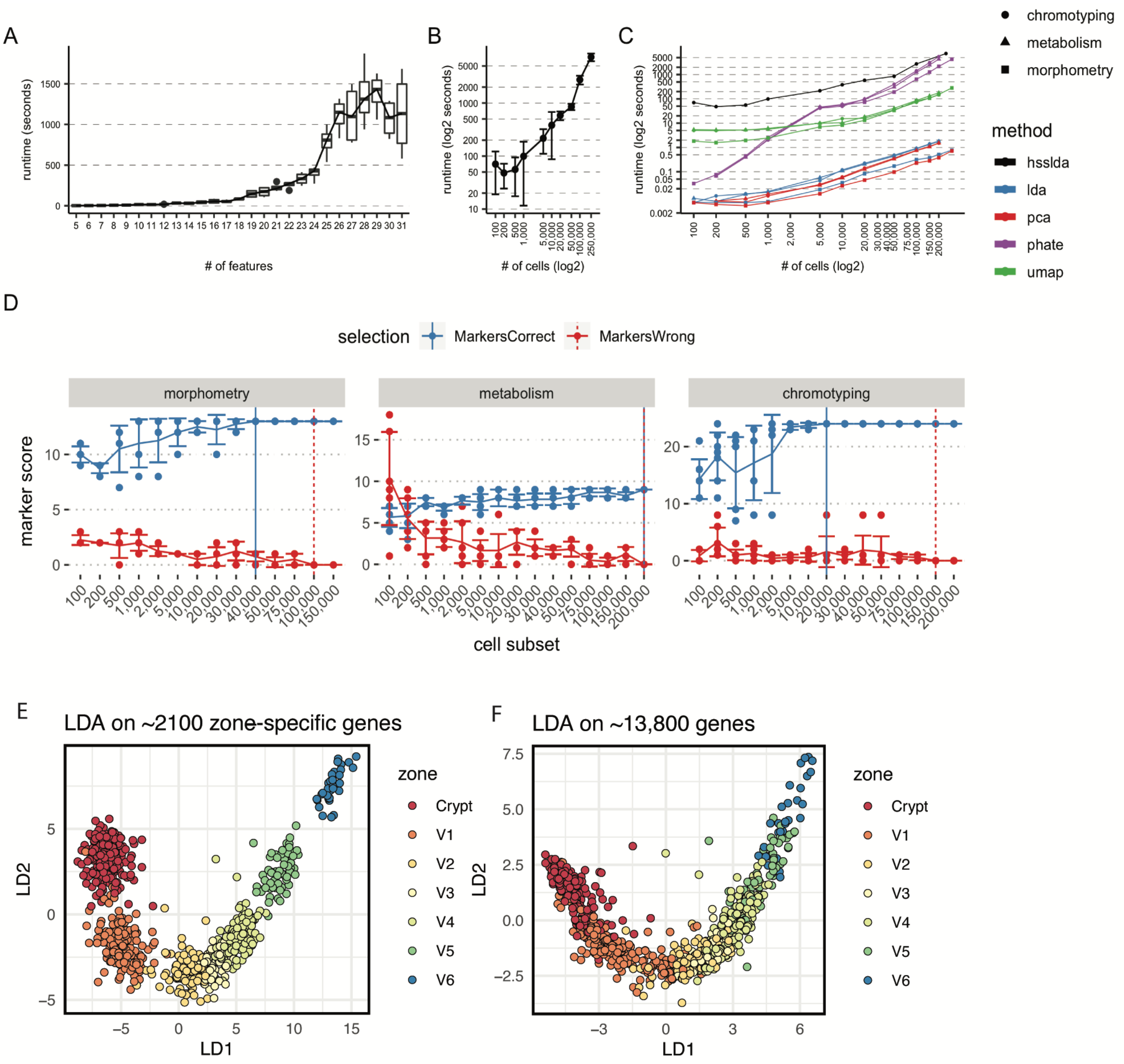

Figure S5: HSS-LDA runtime analysis and assessment of final feature selection.
(A) Runtime analysis for HSS-LDA with varying # of starting markers randomly selected using the same 50,000 cells. **(B)** Runtime analysis with mean and standard deviation for varying # of cells using 32 chromotyping markers as input feature set. **(C)** Runtime analysis of HSS-LDA benchmarked against all other algorithms that do not perform feature selection. **(D)** Assessment of the minimum # of cells required for HSS-LDA to select the feature set that maximally separates class labels. **(E)** LDA directly on the ~2,100 zone-specific genes published by Moor et al., 2018. Runtime was approximately 12.6 seconds. **(F)** LDA directly on the ~13,800 genes after initial preprocessing to remove genes with low variable expression across the dataset. Runtime was approximately ~6.4 minutes.

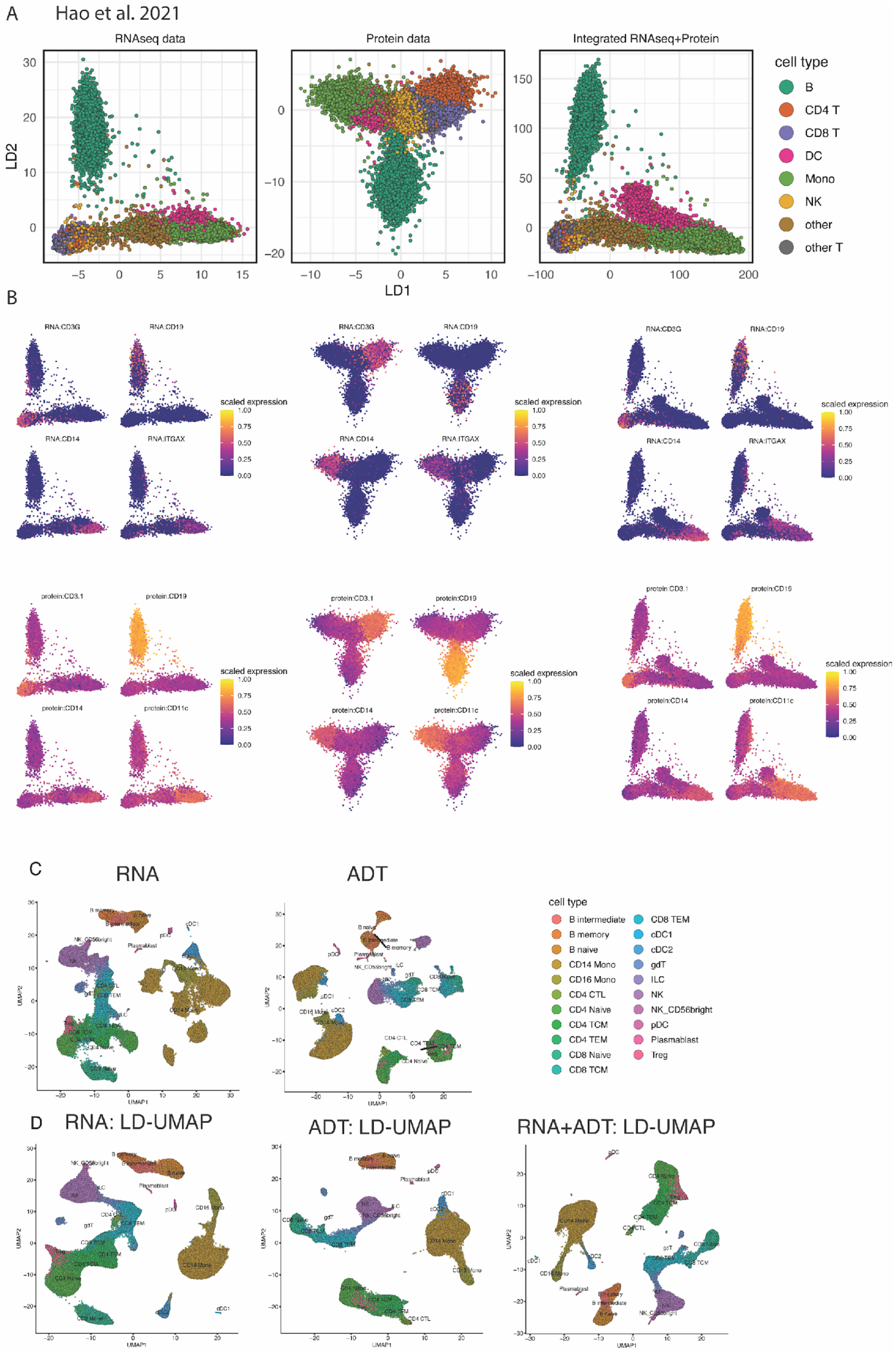
A
Hao et al. 2021
RNAseq data
Protein data
Integrated RNAseq+Protein
LD2
LD1
cell type
B
CD4 T
CD8 T
DC
Mono
NK
other
other T
B
RNA:CD3G
RNA:CD19
RNA:CD14
RNA:ITGAX
protein:CD3.1
protein:CD19
protein:CD14
protein:CD11c
scaled expression
C
RNA
ADT
cell type
B intermediate
B memory
B naive
CD14 Mono
CD16 Mono
CD4 CTL
CD4 Naive
CD4 TCM
CD4 TEM
CD8 Naive
CD8 TCM
CD8 TEM
cDC1
cDC2
gdT
ILC
NK
NK_CD56bright
pDC
Plasmablast
Treg
UMAP1
UMAP2
D
RNA: LD-UMAP
ADT: LD-UMAP
RNA+ADT: LD-UMAP

Figure S6: Supervised dimensionality reduction by LDA for multi-omic data integration
Dimensionality reduction by UMAP or LD-UMAP using multi-omic CITE-seq dataset of 154,491 cells from Hao et al., 2021. **(A)** Biaxial plot of LD1 and LD2 from LDA on PCs 1 to 30 supervised with general immune cell type annotations provided by the original authors for downstream analysis shown with only RNA data *(left)*, only ADT data *(center)*, and both the RNA and protein PCs *(right)*. **(B)** Scaled RNA and protein expression for select gene and protein targets. **(C-D)** Dimensionality reduction by UMAP or LD-UMAP using multi-omic CITE-seq dataset of 154,491 immune cells from Hao et al., 2021. All uwot::umap parameters were the same: n_neighbors = 50, spread = 4, min_dist = 0.1. **(C)** Unsupervised dimensionality reduction by UMAP on RNA (left) and ADT (right) on the first 50 PCs colored by celltype. **(D)** Supervised dimensionality reduction of first 50 PCs by LDA using previously published cell-type annotations determined from RNA or ADT data followed by input of all LDs into UMAP for RNA (left) or ADT (center) data. Multi-omic integration of LDs for RNA and ADT inputted into UMAP (right).

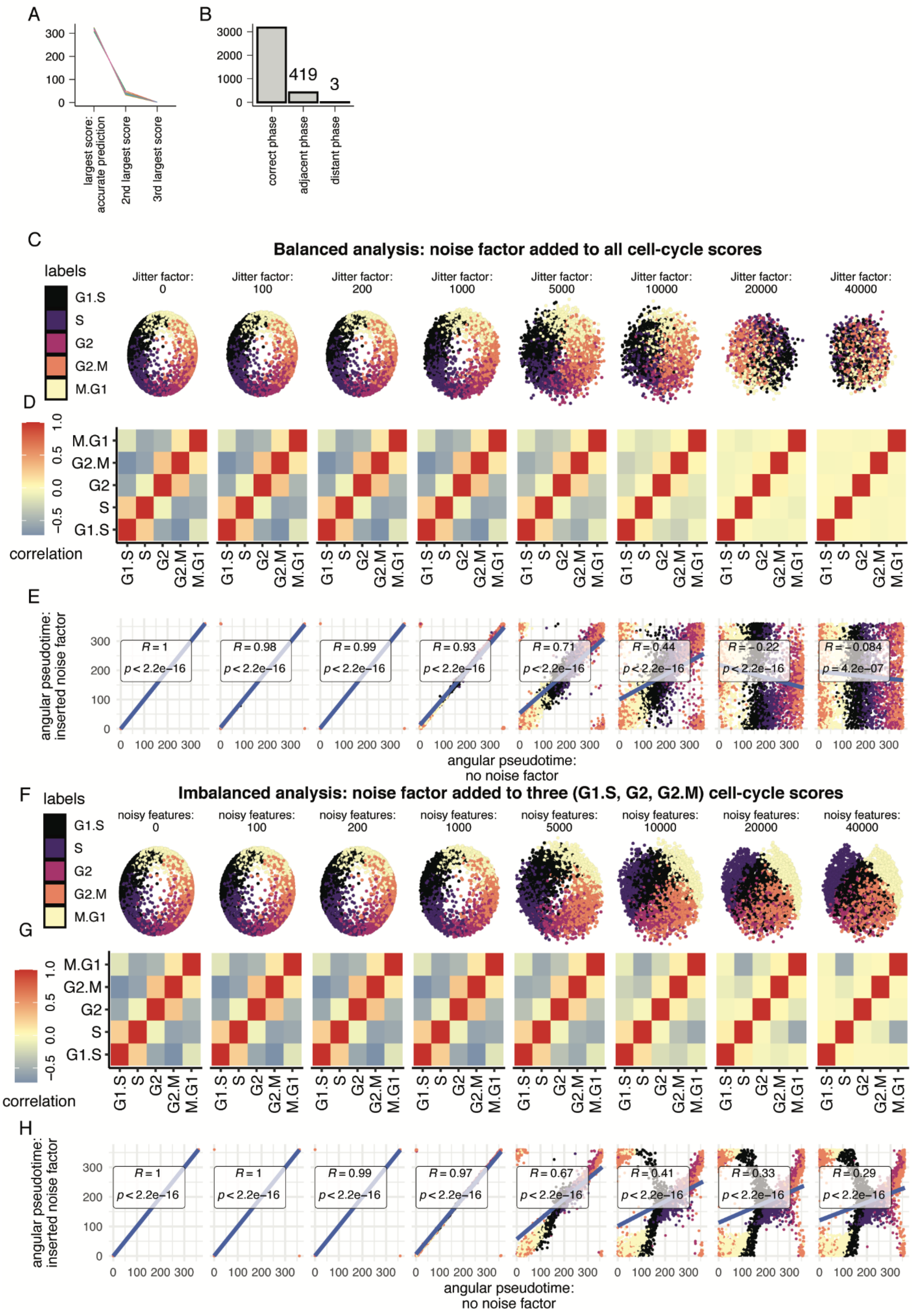
A
B
3000
2000
1000
419
3
largest score: accurate prediction
2nd largest score
3rd largest score
correct phase
adjacent phase
distant phase
C
Balanced analysis: noise factor added to all cell-cycle scores
labels
G1.S
S
G2
G2.M
M.G1
Jitter factor: 0
Jitter factor: 100
Jitter factor: 200
Jitter factor: 1000
Jitter factor: 5000
Jitter factor: 10000
Jitter factor: 20000
Jitter factor: 40000
D
correlation
E
angular pseudotime: inserted noise factor
R = 1
R = 0.98
R = 0.99
R = 0.93
R = 0.71
R = 0.44
R = −0.22
R = −0.084
p < 2.2e−16
p = 4.2e−07
angular pseudotime: no noise factor
F
Imbalanced analysis: noise factor added to three (G1.S, G2, G2.M) cell-cycle scores
noisy features: 0
noisy features: 100
noisy features: 200
noisy features: 1000
noisy features: 5000
noisy features: 10000
noisy features: 20000
noisy features: 40000
G
H
R = 1
R = 1
R = 0.99
R = 0.97
R = 0.67
R = 0.41
R = 0.33
R = 0.29

Figure S7: Cross-validation and noise-insertion analysis shows that cell cycle LDA is an accurate embedding of cell cycle progression and that correlative relationships are essential for constructing circular trajectory models in linear transformation techniques.
(A) Summary barplot of 10-fold cross-validation accuracy results for cyclical LDA model performed on non-overlapping test sets. Accurate cell cycle phase predictions match the largest cell cycle score. 88% test set accuracy. **(B)** Barplot summary of test set predictions indicating cell assignment predictions as either the correct cell cycle phase, an adjacent cell cycle phase, or a distant cell cycle phase. Incorrect predictions are often cells transitioning through the cell cycle in an adjacent cell cycle phase. **(C)** LD embeddings of cell cycle LDAs with increasing amounts of equal noise inserted into all cell cycle scores. **(D)** Correlation heatmaps of cell cycle scores with increasing amounts of equal noise inserted into all cell cycle scores. **(E)** Angular pseudotime estimates of the noisy model versus unperturbed model. A simple linear regression model was generated to determine R2 and P value. **(F)** LD embeddings of cell cycle LDAs with increasing amounts of imbalanced noise inserted into three cell cycle scores (G1.S, G2, G2.M). **(G)** Correlation heatmaps of cell cycle scores with increasing amounts of imbalanced noise inserted into three cell cycle scores. **(H)** Angular pseudotime estimates of the noisy model versus unperturbed model. A simple linear regression model was generated to determine R2 and P value.

Supplementary Table #1:

Algorithm	Version	Availability	parameters
HSS-LDA	0.1	https://github.com/mamouzgar/hsslda	euclidean or PCE score feature selection, specified in figure legends
LDA	MASS: 7.3-51.5	https://cran.r-project.org/web/packages/MASS/index.html	NA
PCA	stats: 3.6.1	Base R package	NA
UMAP	uwot: 0.1.10	https://cran.r-project.org/web/packages/uwot/index.html	default unless otherwise specified in figure legends
PHATE	PHATE: v0.3.0	https://github.com/KrishnaswamyLab/PHATE#r	default unless otherwise specified in figure legends
sparse LDA	sparseLDA: v0.1.9	https://cran.r-project.org/web/packages/sparseLDA/index.html	default unless otherwise specified in figure legends

Table S1: Software versions, accession links, and parameters used for different algorithms.
