# Supplementary material for: Supervised dimensionality reduction for exploration of single-cell data by HSS-LDA
Source: Patterns (N Y). 2022 Jun 24;3(8):100536. doi: 10.1016/j.patter.2022.100536 (PMC9403402; doi:10.1016/j.patter.2022.100536)
Supplement: Document S2. Article plus supplemental information [file mmc2.pdf]

Patterns

Article

Supervised dimensionality reduction for exploration of single-cell data by HSS-LDA

Graphical abstract

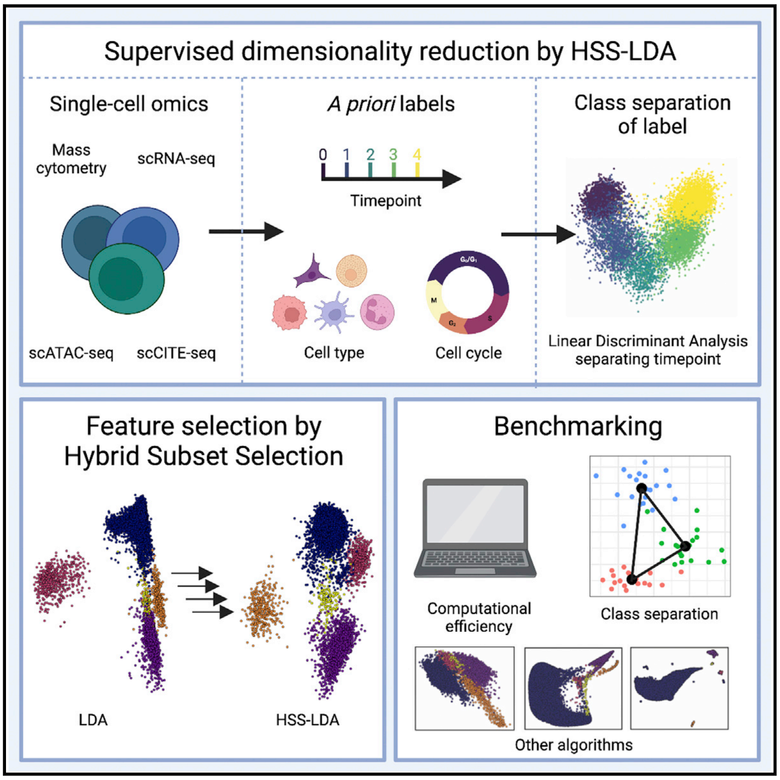

Highlights

- LDA is repurposed for supervised dimensionality reduction of single-cell data
- HSS provides additional feature selection functionality
- Performance is benchmarked against other dimensionality-reduction methods
- LDA can be applied to any single-cell omics data

Authors

Meelad Amouzgar, David R. Glass, Reema Baskar, ..., Albert G. Tsai, Felix J. Hartmann, Sean C. Bendall

Correspondence

dglass@fredhutch.org (D.R.G.),
bendall@stanford.edu (S.C.B.)

In brief

Amouzgar et al. repurpose the classification algorithm, LDA, for supervised dimensionality reduction of single-cell data. Paired with HSS for feature selection, LDA creates intuitive visualizations, separating cells from user-defined labels such as experimental time point and cell-cycle phase. LD axes can be extended to unseen data and used as inputs for other algorithms and methods. HSS-LDA is benchmarked against several popular dimensionality-reduction methods and is applied to a diversity of biological problems and single-cell omics.

Amouzgar et al., 2022, Patterns *3*, 100536
August 12, 2022 © 2022 The Authors.
https://doi.org/10.1016/j.patter.2022.100536

CellPress

Patterns

CellPress
OPEN ACCESS

Article

Supervised dimensionality reduction for exploration of single-cell data by HSS-LDA

Meelad Amouzgar,[1,2,7] David R. Glass,[1,2,3,7,*] Reema Baskar,[1,4] Inna Averbukh,[1] Samuel C. Kimmey,[1,5] Albert G. Tsai,[1] Felix J. Hartmann,[1,6] and Sean C. Bendall[1,2,8,*]
[1]Department of Pathology, Stanford University, Stanford, CA, USA
[2]Immunology Graduate Program, Stanford University, Stanford, CA, USA
[3]Present address: Vaccine and Infectious Diseases Division, Fred Hutchinson Cancer Center, Seattle, WA, USA
[4]Present address: Laboratory of Systems Biology and Data Analytics, Genome Institute of Singapore, Singapore, Singapore
[5]Present address: Ionpath, Menlo Park, CA, USA
[6]Present address: Systems Immunology and Single-Cell Biology, German Cancer Research Center (DKFZ), Heidelberg, Germany
[7]These authors contributed equally
[8]Lead contact
*Correspondence: dglass@fredhutch.org (D.R.G.), bendall@stanford.edu (S.C.B.)
https://doi.org/10.1016/j.patter.2022.100536

THE BIGGER PICTURE Dimensionality reduction enables the visualization of high-dimensional single-cell datasets. Typically, these algorithms are unsupervised, disregarding known biological labels such as cell type or experimental time point. LDA identifies linear combinations of predictors that optimally separate these *a priori* labels. Combined with HSS for feature selection, LDA generates interpretable axes for visualization and analysis of single-cell data. LDA is extensible to unseen data and can also be used as an input to other methods, for example, to integrate multi-label data for UMAP visualization.

LDA enables the exploration of different aspects of cellular heterogeneity, even within the same dataset, by tailoring axes to separate specific, user-defined labels. More generally, this work introduces an alternative approach to visualize single-cell data and highlights the abundance of experimental metadata and cellular features that can be exploited for supervised analysis.

1 2 3 4 5 **Production:** Data science output is validated, understood, and regularly used for multiple domains/platforms

SUMMARY

Single-cell technologies generate large, high-dimensional datasets encompassing a diversity of omics. Dimensionality reduction captures the structure and heterogeneity of the original dataset, creating low-dimensional visualizations that contribute to the human understanding of data. Existing algorithms are typically unsupervised, using measured features to generate manifolds, disregarding known biological labels such as cell type or experimental time point. We repurpose the classification algorithm, linear discriminant analysis (LDA), for supervised dimensionality reduction of single-cell data. LDA identifies linear combinations of predictors that optimally separate *a priori* classes, enabling the study of specific aspects of cellular heterogeneity. We implement feature selection by hybrid subset selection (HSS) and demonstrate that this computationally efficient approach generates non-stochastic, interpretable axes amenable to diverse biological processes such as differentiation over time and cell cycle. We benchmark HSS-LDA against several popular dimensionality-reduction algorithms and illustrate its utility and versatility for the exploration of single-cell mass cytometry, transcriptomics, and chromatin accessibility data.

INTRODUCTION

Single-cell technologies have revolutionized our understanding of biology, enabling granular dissection of the cellular heterogeneity present in complex biological samples. A surge of innovative method development has provided researchers with the means to quantify the transcriptome, immunophenotype, chromatin accessibility, clonality, and antigen-specificity

This is an open access article under the CC BY license (http://creativecommons.org/licenses/by/4.0/).

of single cells, in some cases simultaneously.[1–7] Mass cytometry (CyTOF) facilitates the quantification of ~50 parameters on millions of cells in a single experiment, while sequencing-based approaches can measure tens of thousands of features on tens of thousands of cells.[8,9] This deluge of data encompassing a diversity of omics, cell quantities, dimensionalities, and biological samples is not amenable to a single computational pipeline or approach for analysis, but instead requires a range of flexible computational tools to address different biological questions and therefore analytical needs.

Dimensionality reduction facilitates the exploration of these large, high-dimensional datasets by generating a two-dimensional (2D) coordinate system that enables simultaneous visualization of all datapoints in a single biaxial plot that captures the high-dimensional relationships of cells. Principal-component analysis (PCA) performs unsupervised dimensionality reduction by identifying linear combinations of features that maximize variance.[10] While PCA has been applied to high-dimensional single-cell data, non-linear unsupervised methods, such as uniform manifold approximation and projection (UMAP) and potential of heat diffusion for affinity-based transition embedding (PHATE), have been widely adopted for single-cell visualization due to a superior ability to capture local and global structure, while preventing coordinate overlap.[4,11,12] While these algorithms represent powerful tools for computational biology, they may not always be the optimal choice for a given dataset, based on biological question, analysis goal, and/or available computational resources. Furthermore, while these unsupervised methods provide an unbiased view of the data, they cannot use *a priori* knowledge of sample composition to improve the manifold.

Previously, we introduced linear discriminant analysis (LDA) for the visualization of single-cell morphometry data for hematopathology diagnostics driven by previously defined healthy cell classes.[13] LDA is a classification algorithm that identifies linear combinations of features that optimally separate previously determined class labels.[14] LDA is used primarily to predict the class label of new observations, but we instead exploit the inherent dimensionality reduction of the method for visualization and hypothesis generation, rather than classification. Here, we demonstrate that LDA is an effective supervised tool to visualize and organize cells according to *a priori* labels such as cell type, cell-cycle phase, or experimental time point. We implement hybrid subset selection (HSS), a heuristic approach using elements of both forward and reverse stepwise selection, to identify a set of features that enable enhanced separation of these labels. Furthermore, feature selection by HSS for the optimization of class separation combined with data visualization provides users with a visually intuitive and interpretable understanding of key feature drivers underlying the biological source of variation represented by class labels. We compare and benchmark HSS optimized LDA against PCA, UMAP, and PHATE across three mass cytometry datasets and demonstrate its utility and versatility for the visualization of single-cell transcriptomics, epigenetics, and multi-omic profiling. Finally, to empower researchers to apply supervised dimensionality reduction to their own datasets, we introduce our implementation of LDA with feature selection in the R package *hsslda*.

RESULTS

HSS optimizes supervised dimensionality reduction for single-cell visualization

Supervised dimensionality reduction by LDA takes in a matrix of cells (n) and features (p), as well as a list of *a priori* classes (k), to generate a set of k – 1 LDs (Figures 1A and S1A). LDA leverages these class assignments as a response variable to derive the LDs, which are interpretable linear combinations of features that optimally separate cells by their known, user-defined class assignment. These *a priori* labels can be biological features of cells such as cell types, collection time points, cell lines, cell-cycle phases, or other categorical/ordinal features. Traditionally, dimensionality reduction relies on all defined features (p) as inputs. However, to obtain the optimal separation between classes for visualization, the user needs to tune this feature set so that it best separates the class labels in the data. This separate analysis can often be a time-intensive task for biologists. To facilitate improved dimensionality reduction and visualization, we implemented HSS to augment LDA with an automatic feature selection that optimizes class separation in an interpretable manner (Figure 1B). The HSS-LDA algorithm uses a combination forward and reverse stepwise feature selection heuristic, calculating separation scores across many feature subsets, and selects the final set of features that best separates classes for visualization (see methods).

For example, to understand endoderm differentiation patterns, Kimmey et al. collected endoderm cells across five differentiation time points (k = 5) and applied CyTOF to obtain a single-cell matrix of cells and protein markers (see resource availability statement). In addition to this single-cell matrix of protein features, each cell was annotated with its collection time point. By applying LDA and visualizing the first two LDs, cells from different time points were separated across a single biaxial plot (Figure 1C, left). Day 0 separated from the other time points but its relationship to other time points in differentiation was not evident. While LDA alone can visually separate cells according to their class assignments, we applied HSS to identify the combination of features that optimizes this separation, resulting in improved visualization (Figure 1C, right). HSS-LDA improved differentiation time points with an optimized set of features and revealed a continuous trajectory from human embryonic stem cell (hESC) day 0 to day 6 differentiated endoderm-primed cells.

To assess the utility of HSS-LDA for single-cell visualization, we applied LDA, HSS-LDA, and UMAP to three CyTOF datasets from different biological systems and with different visualization needs.[11] Each dataset was assigned a specific name for this paper: "Morphometry," "T cell metabolic regulome," and "Chromotyping" (data not shown).[13,15] These mass cytometry datasets had unique challenges such as significantly imbalanced cell numbers across classes (e.g., cell types), as well as different biological processes to visualize such as discrete, continuous, or cyclical systems. Compared to LDA, the HSS-LDA visualization either qualitatively improved or retained the original separation of class labels in the HSS-LDA embedding for the three datasets despite using a lower dimensional feature matrix (Figures S1B–S1G). HSS-LDA facilitated the separation of cell types in the morphometry dataset using only 13 features from 17 (Figures S1B and S1C), metabolic states of T cells over time using only 8 features from 32

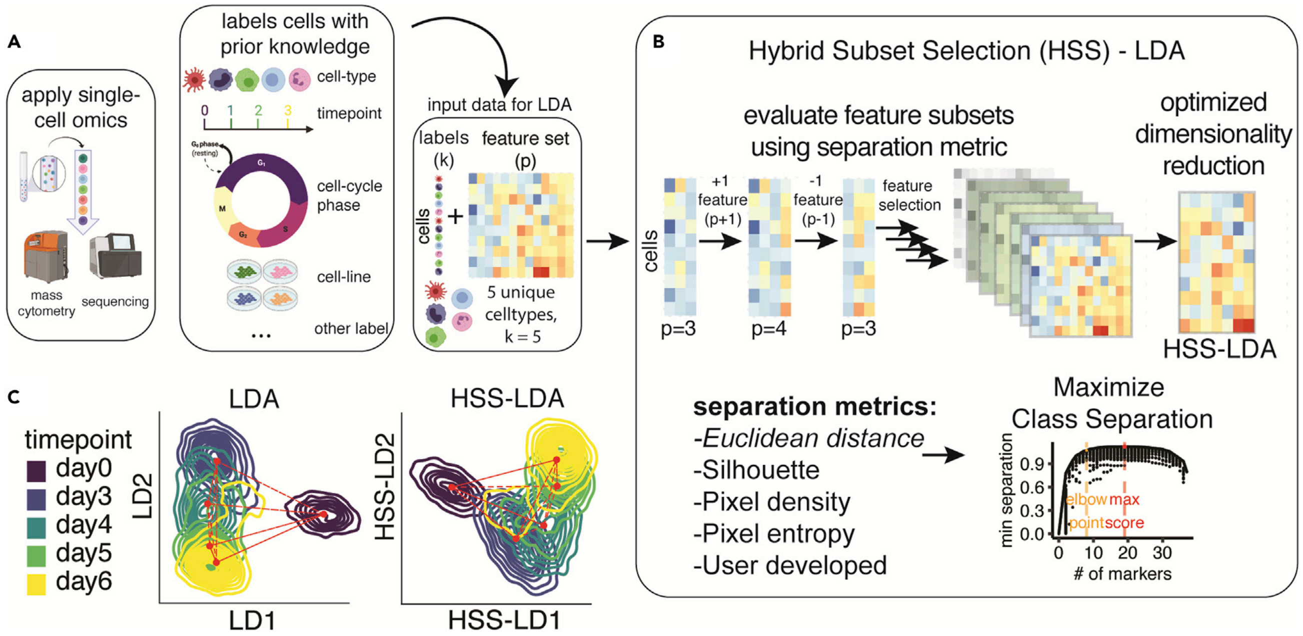

Figure 1. HSS-LDA optimizes dimensionality reduction using feature selection

(A) Workflow demonstrating linear discriminant analysis (LDA) with prior knowledge of class labels of interest for supervised dimensionality reduction and feature selection using hybrid subset selection (HSS).

(B) HSS-LDA performs feature selection to enhance dimensionality reduction and visualization of single-cell data by maximizing class separation via a stepwise feature selection approach, selecting the final model based on a separation metric specified by the user.

(C) Comparison of LDA and HSS-LDA visualization using example endoderm differentiation data.

(Figures S1D and S1E), and cell-cycle phases using only 24 features from 32 (Figures S1F and S1G).

We also explored feature selection through L2 regularization using sparseLDA (sda).[16] To compare performance, we performed LDA, (lda) HSS-LDA, (hsslda), and sda on our three mass cytometry datasets using default settings. Tuning sda parameters did not result in substantially different results (data not shown). We used the same input cells for each algorithm and visualized the first two discriminants (Figures S1H, S1J, and S1L). While all of the approaches rendered useful visualizations that separated classes for each dataset, HSS-LDA provided better separation of blasts from neutrophils and lymphocytes in the Morphometry dataset. This is likely due to our implementation of HSS, which specifically rewards visualizations with the greatest separation of the least-separated groups, enriching for plots that separate all of the classes. Furthermore, in sparseLDA, regularization is performed using the L2 penalty (no L1 implementation is currently available), which drives feature coefficients close to zero, but still retains all of the features in the final model (Figures S1I, S1K, and S1M). HSS explicitly removes features that do not add to class separation, which improves interpretability. Feature weighting was similar between sparseLDA and HSS-LDA in the Metabolism dataset, while more substantial differences arose in the Morphometry and Chromotyping datasets. While sda proved to be a viable tool for single-cell visualization, we focused on HSS-LDA in this paper for the reasons outlined above.

HSS enriches features useful for separating multiple classes. In the Chromotyping dataset, pHH3_S10 expression defines mitotic cells. LDA selected this marker to separate these cells along LD1, but in doing so, obscured other features selected by HSS-LDA that also delineate mitotic cells, such as H3K27ac and H4K16ac (Figures S1F, S1G, and S2E). Furthermore, these markers have the additional value of being differentially expressed in other cell-cycle phases. We observed a similar phenomenon in the Morphometry dataset, in which LD2 in LDA was dominated by CD14, which uniquely marks monocytes (Figures S1B, S1C, and S2A). HSS-LDA instead selected CD45 as a dominant feature of LD2, a marker that is uniquely high in monocytes, absent in erythroids, and moderately expressed in other cell populations. Thus, in addition to identifying an optimized minimum feature set through feature selection to reduce the feature space, the HSS-LD coefficients also provide interpretability of key drivers of class separation in both magnitude and direction, which can be used to guide other analyses (Figure S3A).

HSS-LDA reconstructs both discrete and continuous biological processes

The Morphometry dataset uses a set of markers called scatterbodies that capture immune cellular identities based on structural features that are consistent even in malignancy, thereby discriminating immune and hematopoietic cells extracted from bone marrow (Figure 2A). There are often drastic cell-type imbalances in human tissues, exemplified in the Morphometry dataset by a predominance of neutrophils compared to other cell populations in the bone marrow (Figures 2B and 2C). While this can be re-balanced through equal sampling of each cell type, subsetting the data potentially discards valuable cell information reliant on prior system knowledge. UMAP spreads data to avoid coordinate overlap, but in this imbalanced dataset, the result is

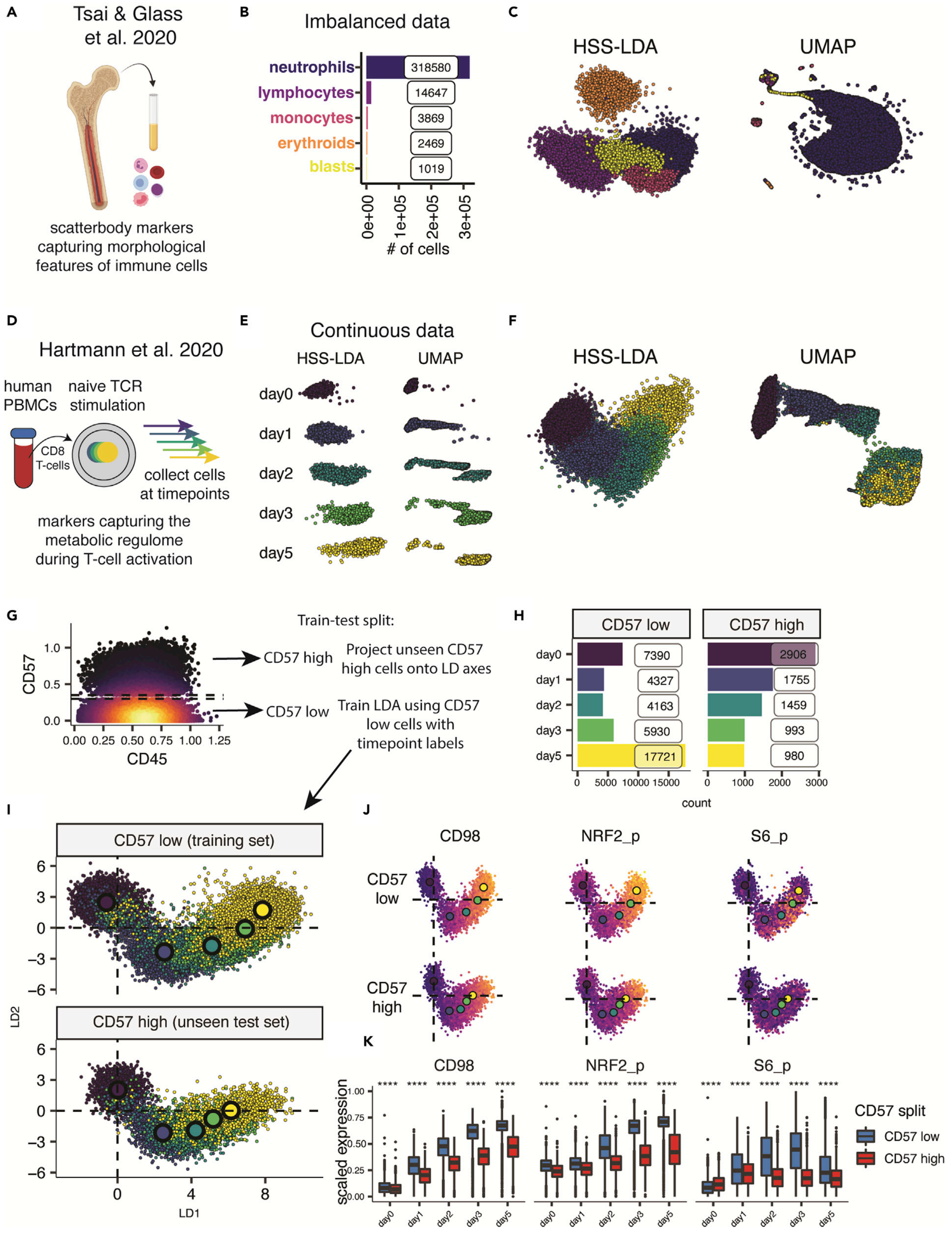

(legend on next page)

neutrophils dominating the entire manifold, making it difficult to see differences between classes. HSS-LDA treats discrete cell types equally and separated cell types regardless of their cell abundance, while preserving cellular relationships by protein abundance (Figures 2C, S2A, and S2B). To test the function of HSS-LDA as a classification algorithm, we trained HSS-LDA for both visualization and classification of discrete cellular identities using cells only from healthy donors (Figure S3B). We visualized HSS-LDA plots and accuracy metrics, finding that HSS-LDA accurately predicted cellular identities of cells derived from patients with hematopoietic malignancies, with a median accuracy of ~90% (Figures S3C and S3D). Thus, both the visualization and classification aspects of HSS-LDA bear utility for biological applications.

The T cell metabolic regulome dataset includes a feature set of markers that capture the metabolic state of human peripheral blood mononuclear cell (PBMC)-derived CD8$^+$ T cells collected across multiple time points after *ex vivo* T cell receptor (TCR) stimulation (Figure 2D). Hartmann et al. demonstrated that markers for metabolic regulation capture the continuous trajectory of metabolic cellular states over time.[15] Cells were labeled by their experimental time point and separated by HSS-LDA, facilitating the visualization of the linear trajectory of T cell activation using ordinal labels. Both HSS-LDA and UMAP separated cells across each time point (Figures 2E, 2F, S2C, and S2D), but LDA uniquely provided both (1) interpretable feature coefficients across each linear discriminant, and (2) facilitated the projection of unseen data onto previously trained LD axes for exploratory analysis. We demonstrated this utility by stratifying T cells by CD57 expression, a marker of senescence and terminal differentiation (Figures 2G and 2H). We trained LDA on CD57^{low} cells and visualized both the linear trajectory of these cells and the respective linear combination of coefficients in a biaxial plot to generate a latent space representation of the metabolic progression of a non-senescent T cell during TCR stimulation (Figures 2I and S3E–S3G). To visualize the metabolic progression of senescent T cells compared to non-senescent T cells during TCR stimulation, we projected the unseen CD57^{high} cells onto the CD57^{low} LD axes and found that CD57^{high} cells had a stunted metabolic progression starting between days 1 and 2 of TCR stimulation compared to CD57^{low} cells (Figure 2I). The mean coordinates of CD57^{high} cells at day 5 overlapped on the manifold with the CD57^{low} cells at approximately days 2–3 of TCR stimulation, reflecting the idea that these cells shared a common metabolic state on different days of TCR stimulation (Figure 2J). Notably, the three features that contributed most to HSS-LD1, were significantly differentially expressed between CD57^{low} and CD57^{high} cells at all time points (Figures 2J, 2K, and S1E). These differences were not an artifact of test/train sampling (Figures S3H and S3I). We explicitly assessed all of the metabolic markers and implemented LDA rather than HSS-LDA to train a more metabolically integrative model that is less biased toward a CD57^{low}-specific feature set. Through supervised dimensionality reduction by LDA, we show that a supervised method can be trained on a baseline cellular state such as T cells with healthy proliferation potential, and distinct cellular states such as those with senescent or terminally differentiated phenotypes can be compared to a baseline state while integrating high-dimensional data in a visually intuitive manner. These results demonstrate that LDA and HSS-LDA can be applied to datasets with both categorical and ordinal labels for visualization and interpretation of discrete and continuous single-cell biological systems.

HSS-LDA captures cyclical biological processes within multi-label data

Cellular division is an important and highly regulated biological process that maintains tissue homeostasis with cellular turnover and can become corrupted in malignancy. Through cellular division, global chromatin structure undergoes significant changes to facilitate DNA replication and separation into two cells. To better understand the dynamics of chromatin structure regulators through the cell cycle, we applied HSS-LDA to highly multiplexed chromatin content data from single cells (i.e., chromotype: a collection of chromatin-modifying factors and histone modifications) across cell lines and cell-cycle states (Figures 3A and 3B). Global chromatin content as defined by single-cell abundance of chromatin-modifying factors and histone modifications capture the distinct, endogenous epigenetic patterning of different cell lines as well as the expression patterns of these markers across the cell cycle (Figure 3A). This dataset is particularly unique because it contains (1) two sets of labels (cell type and cell cycle), and (2) a cyclical biological process (cell cycle), resulting in five cell lines and five cell cycle phases (Figure 3B). Both the cell lines

Figure 2. HSS-LDA reconstructs both discrete and continuous biological processes and can embed new, unseen cells onto the visualization for exploratory analysis

(A) Conceptual diagram of immune cells extracted from healthy bone marrow and stained using morphometric markers for CyTOF.

(B) Bar plot summarizing imbalanced class distribution of immune cell populations.

(C) Comparison of HSS-LDA using pixel class entropy (PCE) score for feature selection and UMAP demonstrating discrete class visualization using the same input cells.

(D) Conceptual diagram of human CD8 naive T cells extracted from PBMCs for *ex vivo* TCR stimulation, collected on days 0–5 of activation, and stained with metabolic markers for CyTOF analysis.

(E) Comparison of HSS-LDA using Euclidean distance for feature selection and UMAP demonstrating a linear trajectory using the same input cells faceted across each time point. The number of cells is balanced across each time point. UMAP implemented with published settings: n_neighbors = 15 and min_dist = 0.02.

(F) Unfaceted HSS-LDA and UMAP plot of (E).

(G) Biaxial CD57 versus CD45 plot colored by density, showing train-test split for stratification CD57^{low} and CD57^{high} cells. HSS-LDA is trained on CD57^{low} cells and the unseen CD57^{high} cells are used as a test set projected onto the CD57^{low} LD embedding.

(H) Bar plot summary counts for CD57^{low} and CD57^{high} training and test sets.

(I) Biaxial LD plots of CD57^{low} cells and embedded CD57^{high} cells labeled with the centroid point for each time point.

(J) Protein expression of biaxial HSS-LD plots for 3 example markers: CD3, CD98, and MCT1.

(K) Boxplot summary of protein expression for CD57^{low} and CD57^{high} cells across each time point. Wilcoxon signed-rank test performed between CD57^{low} and CD57^{high} cells across each time point. *$p \leq 0.05$; **$p \leq 0.01$; ***$p \leq 0.001$; ****$p \leq 0.0001$.

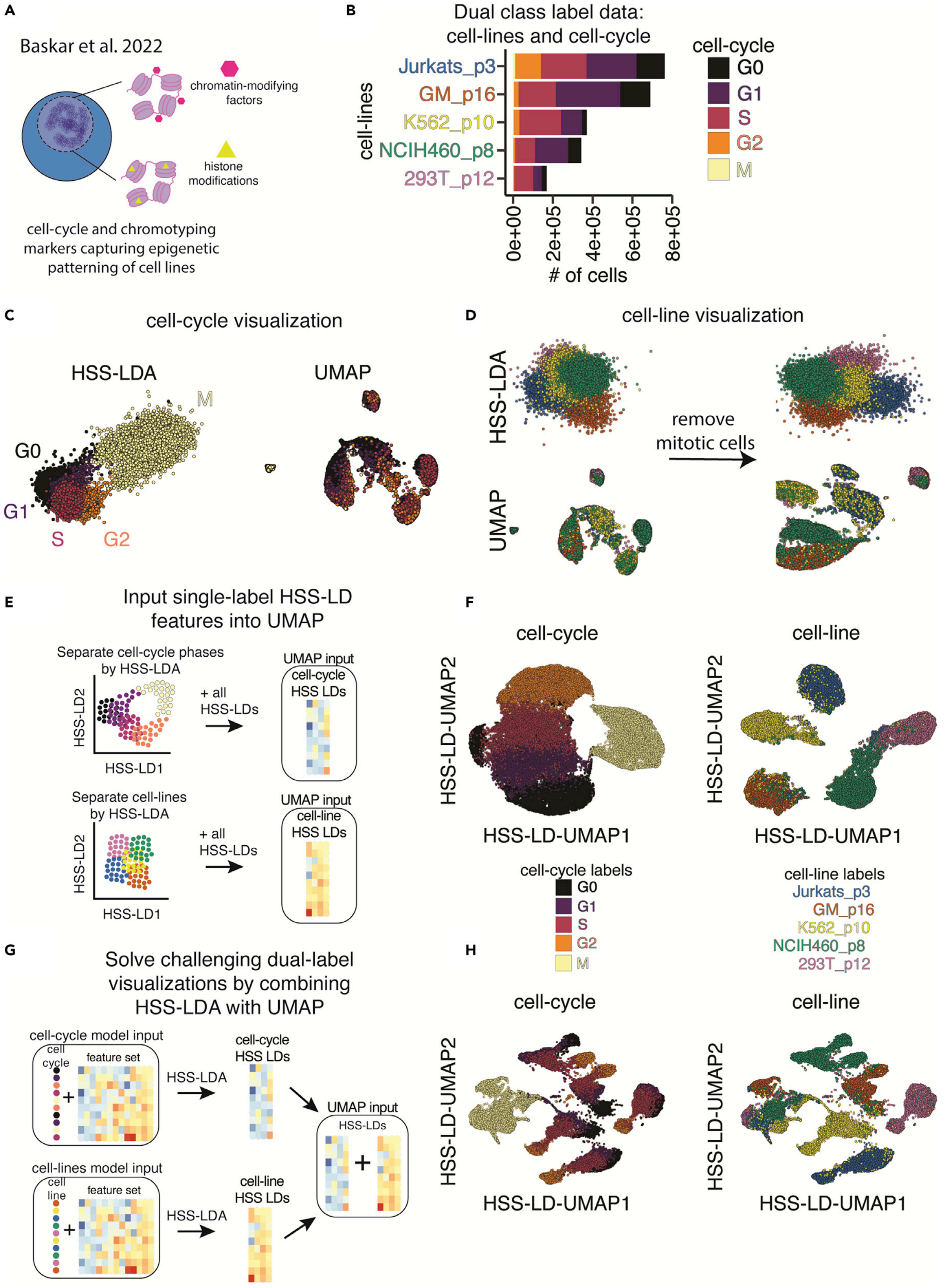

(legend on next page)

CellPress
OPEN ACCESS

and cell-cycle phases contain an imbalanced distribution of cells. Ground truth cell-cycle phases were labeled by manual gating of CyclinB1, IdU, phosphorylated H3, and pRB.[17,18]

The cell cycle is thought of as a circular process in which the M phase parental cell divides to form two G0/G1 child cells. Here, we wanted to explicitly visualize this cyclical trajectory to track protein expression across the cell cycle independent of cell line heterogeneity. We ran HSS-LDA and UMAP using the same initial set of epigenetic markers and trained HSS-LDA using the cell-cycle labels. HSS-LDA successfully separated the cell-cycle states and captured the circular trajectory of the cell cycle, whereas UMAP did not separate cell-cycle labels as an adequate representation of the cell-cycle stages (Figures 3C, S2E, and S2F). The UMAP manifold was confounded by the imbalanced distribution of the dataset and attempted to capture information unique to both the cell lines and cell cycle. HSS-LDA selected features that separate cell-cycle labels and generated an interpretable linear combination of features that separates these cell labels (Figures S2E and S2F). UMAP would require manual intervention to identify the feature subset to adequately visualize the cell cycle, and even then, the cell-cycle signal may still be confounded by distinct cell line properties that may require signal correction methods to deconvolve cell-cycle and cell line properties. The mitotic cells were hidden in the UMAP, and other cell-cycle phases were projected onto multiple areas of the manifold. While parameter tuning could perhaps improve the UMAP embedding, it cannot fully resolve these visualization challenges. HSS-LDA takes advantage of prior knowledge of the cell-cycle labels and feature selection captures the relevant cell-cycle information independent of the cell lines, sufficiently visualizing the cyclical trajectory using a linear transformation. Here, we can visibly see marker transitions of puromycin and CyclinB1 protein abundances along the cell-cycle trajectory (Figure S2E). By visualizing cellular relationships in a manner that reflects the cyclical trajectory of the cell cycle, we can more intuitively study the markers that directly contribute to cell-cycle dynamics but also independently study the patterns of other protein markers that are not used to construct the cell-cycle embedding.

At the same time, within the same dataset, HSS-LDA could reveal cell line differences, visualizing them independent of cell-cycle phases. While projecting the same data using cell line labels proves more difficult for both HSS-LDA and UMAP, the HSS-LDA plot further improved when mitotic cells were removed (Figures 3D, S2G, and S2H). However, this remains a challenging biaxial visualization task for both HSS-LDA and UMAP, which is one of the current gold standard dimensionality-reduction methods for visualization. For both cell cycle and cell line, low-abundance classes (e.g., mitotic cells) were better visualized with HSS-LDA as compared to UMAP (Figures 3C and 3D), because they occupy a proportionally larger area on the manifold in UMAP, as previously shown with the Morphometry dataset. Thus, HSS-LDA can be used to visualize cyclical biological trajectories and discrete cellular identities with heterogeneous data distributions, enabling the identification of features uniquely associated with either cell-cycle or cell line classes.

HSS-LDA as UMAP input integrates variance from multiple class labels into a single visualization

While we used HSS-LDA as a visualization tool with the first two LDs, HSS-LDA produces multiple LDs. The number of LDs produced in the model is calculated as the [# of classes − 1]. Subsequent HSS-LDs contain additional information that separate classes of interest (Figure S1A). To capture data patterns resulting from more than one source of known variance, we hypothesized that HSS-LDs generated from multiple class labels could be used as input to unsupervised dimensionality-reduction methods. We performed supervised dimensionality reduction by HSS-LDA for either cell-cycle or cell line labels and input the HSS-LDs as features into UMAP to generate an HSS-LD-UMAP embedding that sufficiently separates classes (Figures 3E and 3F).

Given that HSS-LD-UMAP embeddings can separate classes within each single label, we tested whether we could exploit a combination approach to dimensionality reduction to visualize both cell line and cell-cycle class labels in a single biaxial plot. We combined the HSS-LDs from the two separate HSS-LDA analysis for cell line and cell cycle into a single table and input this as a feature set into UMAP to generate a combinatorial HSS-LD-UMAP embedding (Figure 3G). The resulting biaxial plot preserves both cell line and cell-cycle relationships in a biologically meaningful manner (Figure 3H). Cell lines cluster separately (Figure 3H, right panel) while still preserving the cell-cycle trajectory from the G0–G2 state within each cell line (Figure 3H, left panel).

Cell line differences are more distinct than cell-cycle differences, with major patterns being driven by basal epigenetic differences between cell lines. As expected, unlike all other cell-cycle states, the global chromatin content of cells in the mitotic phase is highly conserved across cell lines. As shown, dual-label visualizations can be useful to demonstrate distinct

Figure 3. HSS-LDA reconstructs cyclical biological trajectories and can be input as features into UMAP to solve challenging dual-class visualization tasks

(A) Conceptual diagram of cell-cycle and chromotyping markers of various cell lines for CyTOF analysis.

(B) Bar plot summary of cell counts for each cell line in various cell-cycle phases.

(C) Comparison of HSS-LDA using Euclidean distance for feature selection and UMAP visualizing the cell cycle.

(D) Comparison of HSS-LDA using Euclidean distance for feature selection and UMAP both including and excluding mitotic cells to visualize cell lines.

(E) Conceptual diagram demonstrating prior supervised dimensionality reduction using HSS-LDA to initialize UMAP.

(F) HSS-LDA-initialized UMAP plots of the cell-cycle and cell line labels. UMAP parameters were selected qualitatively; for cell cycle: n_neighbors = 25, spread = 7; for cell lines: n_neighbors = 15, spread = 1.

(G) Conceptual diagram demonstrating prior supervised dimensionality reduction using HSS-LDA to initialize UMAP for dual-class labeled data visualization. HSS-LDA is computed separately on cell-cycle and cell line labels, and the HSS-LDs are merged as the feature set input to initialize UMAP.

(H) HSS-LDA-initialized UMAP plots demonstrating dual-class visualization of both cell line and cell-cycle systems in a single biaxial plot. UMAP parameters were selected qualitatively: n_neighbors = 10, spread = 4.

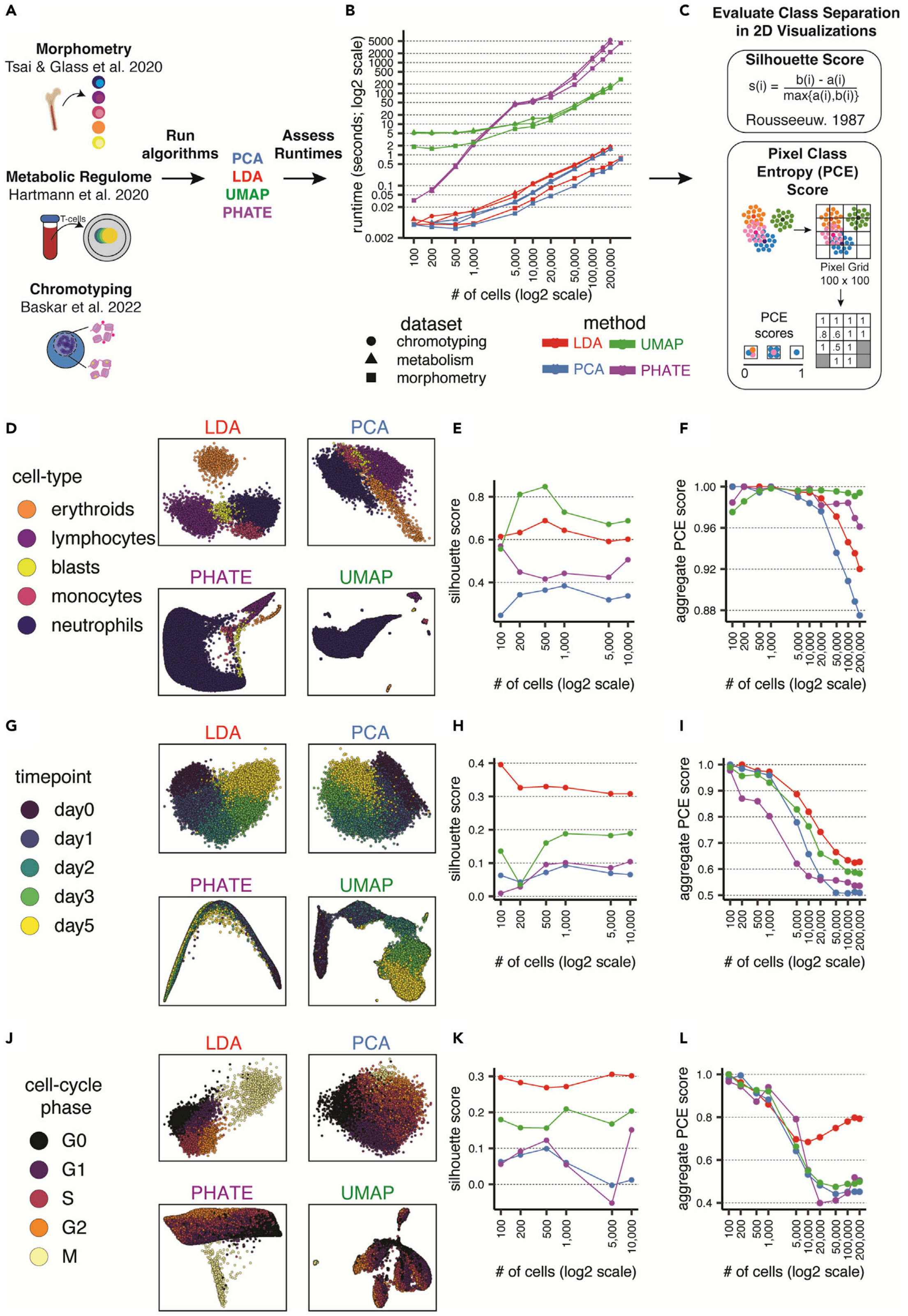

Figure 4. LDA is computationally efficient and scalable and adequately separates class labels

(A) Conceptual diagram for comparing various dimensionality-reduction algorithms. PCA, LDA, UMAP, and PHATE algorithms are applied to 3 CyTOF datasets, and runtimes are assessed to determine efficiency and scalability of the algorithm.

(legend continued on next page)

patterns in a biological process across different systems. Apart from better representing multiple known sources of heterogeneity in a single embedding, prior supervised dimensionality reduction by LDA also significantly reduces the number of features input into UMAP from a full panel of markers to a smaller set of HSS-LDs, reducing UMAP runtime on large single-cell datasets. This is similar to one of many advantages when performing PCA to reduce the feature set before UMAP in high-dimensional genomics datasets. Thus, HSS-LDA for supervised dimensionality reduction can be used in advance of unsupervised methods such as UMAP to help solve challenging dual-label and multiclass visualization tasks in a single embedding.

Benchmarking LDA efficiency and class separation against common dimensionality-reduction algorithms

To further assess the utility of LDA and HSS-LDA, we extended our comparison to other popular dimensionality-reduction methods, including PCA, UMAP, and PHATE across the three mass cytometry datasets (Figure 4A).[10–12] PCA is an ideal comparison because it is conceptually similar to LDA in that they both are linear transformation techniques. While PCA finds directions of maximal variance, LDA finds the feature subspace that maximizes class separability. UMAP is a non-linear, dimensionality-reduction technique that is arguably the most popular single-cell visualization tool, and computationally shares many similarities with its related predecessor tSNE.[19] UMAP is a hybrid dimensionality-reduction approach, as UMAP is initialized using a spectral embedding of the normalized Laplacian eigenmap (LE), which is important for its retention of global structure. PHATE, more recently introduced, is an information-geometric distance approach to capture local and global non-linear structure for dimensionality reduction. PHATE is also a hybrid approach, as it uses multidimensional scaling (MDS) in the final embedding.

We compared LDA to these diverse algorithms to emphasize use cases in which one algorithm may be more suitable than another. To fairly compare runtimes, we used the same feature set and input cells for each algorithm. As anticipated, LDA and PCA were significantly faster algorithms than UMAP and PHATE. We emphasize that a log2 scale was required to adequately visualize the runtime discrepancy between the algorithms as cell counts increased (Figure 4B). LDA is non-stochastic, reproducible, and robust with even low cell counts. Visualizing all four algorithms across the varying cell counts demonstrated that cells will generally occupy the same phenotypic coordinates in the LD embedding when using LDA (Figures S4A–S4C). The scalability of LDA makes it amenable for feature selection using our hybrid subset selection approach. HSS-LDA runtimes converge with other dimensionality-reduction methods that do not provide feature selection when reaching a range of cells that can accurately identify a minimum feature set (Figures S5A–S5C). Using the three mass cytometry datasets, we identified a heuristic of approximately 50,000–200,000 cells to be used as a subset before running HSS-LDA to identify an optimized feature set, although there will be variability with every dataset (Figure S5D). Once the HSS-LDs are computed, the model can non-stochastically project the same data to reproduce the exact same axes or project unseen data within seconds, as was done in the T cell metabolic regulome dataset training the LDA model on the CD57^{low} cells and projecting the CD57^{high} cells onto the same LD axes for rapid dimensionality reduction (Figures 3G–3K).

One of the goals of HSS-LDA is to provide biologically interpretable 2D axes that facilitate exploration and visualization of features and cellular relationships underlying the separation of labeled classes. The interpretability of LDA is therefore predicated on the ability of the algorithm to separate labeled single-cell data. To quantitatively assess class label separation, we varied cell count inputs into each algorithm and applied two separation metrics: (1) silhouette score and (2) pixel class entropy (PCE) score (Figure 4C). Silhouette score is a measure of how similar cells are to their own cluster compared to other clusters by accounting for both intra-cluster and inter-cluster Euclidean distance of each class. PCE score pixelates the biaxial plot into a grid and computes an average PCE score measured by the entropy of all class labels in each pixel of the grid; the approach is further described in the methods. To fairly assess the four algorithms, we used the features (not LDs) selected by HSS as input into PCA, UMAP, PHATE, and LDA. In this way, each algorithm sees the identical input matrix of observations and features.

A 50,000-cell subset of the three mass cytometry datasets and their respective labels (Figures 4D, 4G, and 4J, left) were embedded using these methods. We found that LDA adequately separated class labels across the three datasets and often performed better compared to other dimensionality-reduction algorithms. Silhouette score summaries related that LDA performed second best to UMAP in separating cell types in the Morphometry dataset, although as demonstrated in Figure 2, UMAP failed to handle data imbalances and yielded a less useful visualization due to underrepresentation of rare cell types. LDA performed the best separating intra-cluster and inter-cluster distance across time points in the T cell metabolic regulome, and cell-cycle phases in the Chromotyping datasets compared to the other algorithms (Figures 4E, 4H, and 4K, center).

When comparing PCE scores in Morphometry, UMAP and PHATE performed better than LDA, particularly at the highest cell inputs (Figures 4F, 4I, and 4L, right). However, in the less discretized, more continuous T cell activation and cell-cycle datasets, PCE scores for LDA were superior. We concluded that LDA is suitable for visualizing diverse biological systems, is robust to data imbalances, and may be a preferred dimensionality-reduction algorithm depending on the visualization needs

(B) The average runtime of 3 analyses across 3 datasets for each algorithm are shown across different dataset sizes on a log2-transformed scale. Default algorithm settings are used.

(C) Summary of silhouette score and PCE score to assess separation of class labels of interest for each algorithm. Both metrics can be used for feature selection by HSS-LDA.

(D–L) Summary plots of each algorithm applied to the morphometry, T cell metabolism, and chromotyping datasets. (Left: D, G, and J) Representative biaxial visualizations of each algorithm using 10,000 cells. (Center: E, H, and K) Average silhouette score across different cell counts for each algorithm. (Right: F, I, and L) Average PCE score in a 100 × 100 pixel grid across different cell counts for each algorithm.

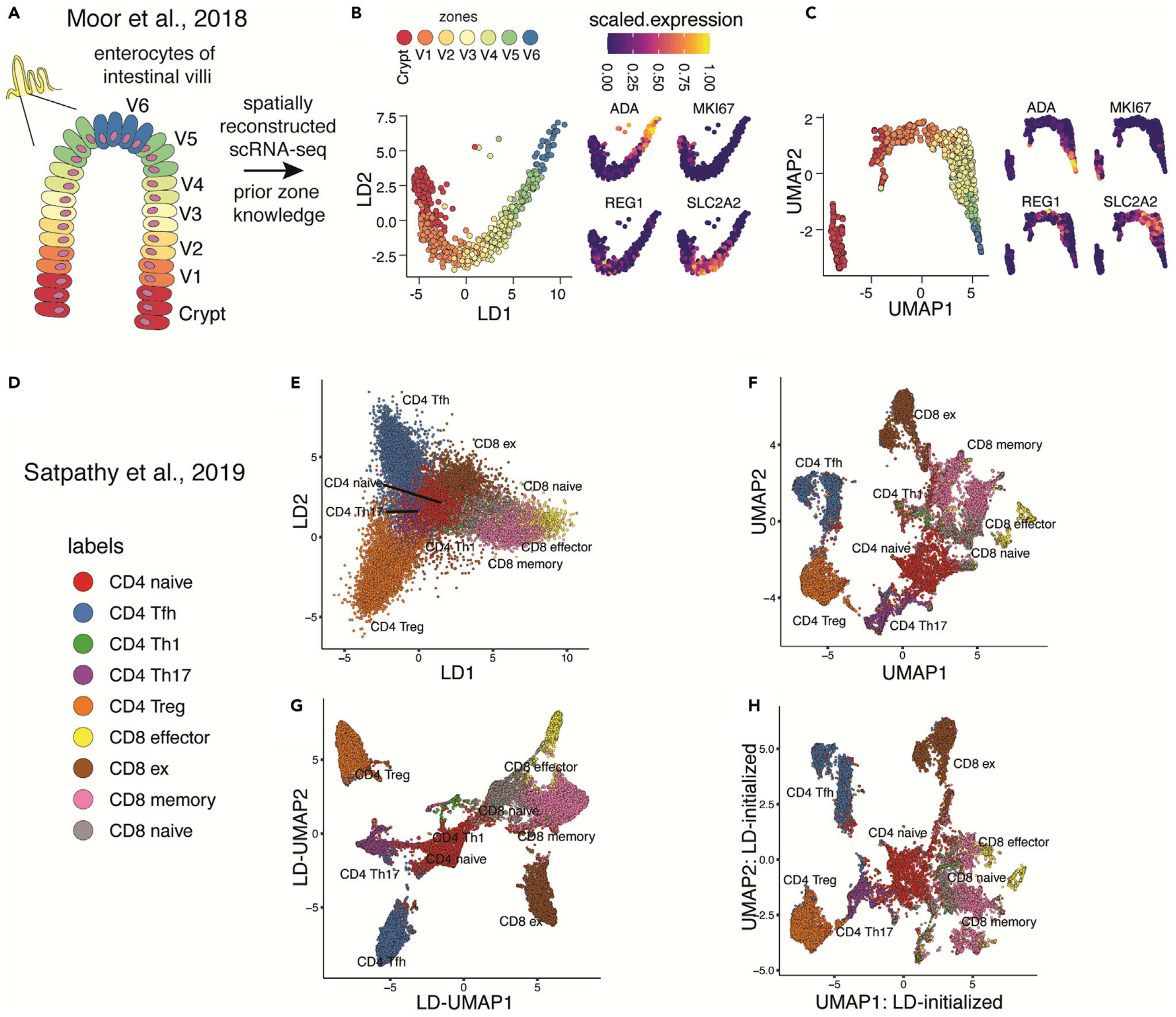

Figure 5. LDA utility extends to single-cell sequencing data to reconstruct linear trajectories as well as organize single-cell chromatin accessibility data using semi-supervised dimensionality reduction

(A–C) Dimensionality reduction using a single-cell dataset of enterocytes of the intestinal villi from Moor at al.[20] (A) Conceptual diagram for (B) and (C) showing enterocyte differentiation from the crypt and across the intestinal villi with prior intestinal zones identified using spatial transcriptomics. (B and C) Comparison of LDA and UMAP demonstrating the linear trajectory of enterocyte differentiation paired with scaled expression of key genes.

(D–F) Dimensionality reduction using single-cell ATAC chromatin accessibility data of T cells from Satpathy et al.[21] (D) Cell-type labels color key. (E) LDA embedding supervised with prior known cell-type labels. (F) UMAP embedding of the same feature set input in (E).

(G) UMAP embedding generated from all 8 LDs generated in (E) input into UMAP.

(H) UMAP embedding initialized by the first 2 LDs (from E) for semi-supervised dimensionality reduction.

when *a priori* knowledge of a class label is known, particularly in ordered and continuous datasets.

LDA captures the trajectory of enterocyte differentiation by single-cell transcriptomics

Given the particular performance of LDA and HSS-LDA in summarizing ordered progressions in single-cell mass cytometry data, we asked whether it could have utility for a similar process mapped by single-cell RNA sequencing (scRNA-seq) as well. We applied supervised dimensionality reduction by LDA to a spatially reconstructed scRNA-seq dataset of enterocytes of the intestinal villi (Figure 5A). Moor et al. used spatial transcriptomics to identify gene sets that corresponded to the differentiation patterns of enterocytes across the intestinal villi, then used these validated gene sets to generate a spatially reconstructed scRNA-seq dataset of enterocytes with zone labels that correspond to their location and differentiation state.[20] To test whether LDA could reconstruct the linear trajectory of enterocyte differentiation, we took the prior zone labels and the first 50 principal components as the input feature set into LDA. LDA improved the linear trajectory

visualization of cells in a biologically relevant manner when compared to UMAP (Figures 5B and 5C). We found that expression of landmark genes with distinct spatial patterns across the intestinal villi were reflected in the arrangement of cells in LDA (Figures 5B and 5C).[20] Adenoside deaminase (ADA) at the villus tip and SLC2A2 mid-villus demonstrated biologically relevant expression patterns that were less coherent in the UMAP, but were preserved with LDA. While prior processing of sparse scRNA-seq data typically involves PCA before further dimensionality reduction and analysis, LDA directly on ~2,100 zone-specific genes and ~13,800 genes produced results similar to LDA on the first 50 principal components (Figures S5E and S5F). These findings demonstrate the utility of LDA for visualization of scRNA-seq data.

LDA organizes T cell heterogeneity seen by single-cell chromatin accessibility analysis

To extend the diversity of single-cell sequencing data types that LDA could be applied to, we also investigated its utility on a single-cell assay for transposase-accessible chromatin with sequencing (scATAC-seq) dataset of CD4 and CD8 T populations.[21] Here, T cell populations include naive, memory, helper, effector, and exhausted subsets as sorted and annotated by the original authors. We processed ATAC peaks using the same methods as the original authors. We performed LDA supervised with cell-type labels, and UMAP using the same input matrix. Both LDA and UMAP separated cell-type labels in a biologically meaningful manner (Figures 5D–5F). More quiescent cell states, such as CD4 and CD8 naive T cells, clustered together while effector cell types were more distant in phenotypic space. To test whether a coherent, supervised, non-linear embedding could be generated using scATAC-seq data, we used all eight LDs created by LDA to separate cell types as input to UMAP (Figure 5G). As with the Chromotyping dataset (Figures 3E–3H), LD-UMAP facilitated clearer separation of labeled populations than unsupervised UMAP (Figure 5F). Kobak and Linderman previously demonstrated the importance of non-random initialization with UMAP and tSNE to preserve global structure.[19] To test whether LDA could be combined with UMAP for a more subtle, semi-supervised embedding, we initialized UMAP with LD1 and LD2 coordinates (Figure 5H). The LD-initialized UMAP better preserved the global relationships of both the progression to CD8 exhausted states and the interrelation between the different naive cell fractions compared to the spectral-initialized UMAP (Figure 5F). Furthermore, we see separation of CD8 memory cells into three states in LD-initialized UMAP, which underscores the utility of LDA in more than separating known class labels. Thus, supervised dimensionality reduction by LDA can be used as a standalone algorithm or as input to unsupervised methods such as UMAP for latent space representations of scATAC-seq data.

LDA facilitates embedding of integrated multi-omics data

Recently, weighted nearest neighbor (WNN) analysis was introduced to integrate multimodal single-cell data for unsupervised dimensionality reduction by UMAP.[22] Given the utility of LDA for visualization of mass cytometry, scRNA-seq, and scATAC-seq data, we asked whether LDA could also be used to integrate and visualize multi-omic datasets. We curated a published human PBMC dataset of 154,491 cells with cellular indexing of transcriptomes and epitopes by sequencing (CITE-seq) data, which quantifies both RNA transcript abundance and the abundance of antibody-derived tags (ADTs) specific to predetermined surface protein targets.[5,22] Using the cell-type labels provided by the original authors, we performed LDA using RNA data (left), ADT data (center), and the integrated data (right) (Figure S6A). As expected, both RNA and ADT expression patterns of defining lineage markers were appropriately expressed and absent in the relevant cell types (Figure S6B). While UMAP provided a satisfactory visualization using either the RNA (left) or ADT data (right) (Figure S6C), separating cell types by LDA and inputting LDs into UMAP provided enhanced separation of granular immune cell subsets using RNA data (left), ADT data (center), and the integrated data (right) (Figure S6D). In addition, data integration by LDA was computationally efficient—we were not able to compare the integrated LD-UMAP manifold to WNN-UMAP without considerable subsampling of the data due to the substantial memory requirements of WNN on a dataset of this size. We therefore conclude that LDA can be used for the integration and visualization of multi-omic datasets.

Reconstructing scRNA-seq-based cell-cycle pseudotime of activated human T cells using LDA

Given the utility of LDA to capture trajectories in continuous datasets (Figures 2D–2K and 5A–5C) and act as an input for other embedding methods (Figures 3E–3H), we tested whether it could help visualize a continuous, circular biological process in which the classes were based on a score derived for single-cell sequencing information. We curated an *ex vivo* TCR stimulation dataset that we call T cell proliferation tracing.[23] This dataset contains primary human T cells labeled with carboxyfluorescein succinimidyl ester (CFSE), stimulated for 3 days *ex vivo*, and prospectively isolated based on cell division state (i.e., 0 divisions, 1 division, 2 divisions) for scRNA-seq analysis.

To summarize the entire cell-cycle process using LDA, we computed the cell-cycle phase scores for G1.S, S, G2, G2.M, and M.G1 using previously published methods (Figure 6A).[24–26] Cell-cycle scores were calculated using a curated list of genes with known increased enrichment in each phase. A cell was given a score for each phase of the cell cycle, and the phase with the largest score was the assigned cell cycle state of the cell (Figures 6B and 6C). However, these cell-cycle phases are not entirely discrete processes from one another, which was reflected by the correlation seen between adjacent phases, driven by cells transitioning between phases (Figure 6D). At the same time, non-adjacent phases, which had no cells transitioning between them, were anti-correlated. We applied LDA to a matrix of cell-cycle scores and provided the list of cell-cycle labels, resulting in a cyclical LDA visualization that accurately separated the cell-cycle phases according to their expected position (Figure 6E).

To assess whether the model was overfitting to the cell-cycle scores, we performed cross-validation by splitting the dataset into 10 non-overlapping test sets, training the LDA model on the cell-cycle scores, and calculating cell-cycle accuracy. We determined that cell-cycle label predictions were 88% accurate (3,180 of 3,602 cells) (Figure S7A). Of the 422 cells predicted in an inaccurate cell-cycle phase, 419 cells (99.3% of the 422) were predicted to be in an adjacent cell-cycle phase (Figures S7A and

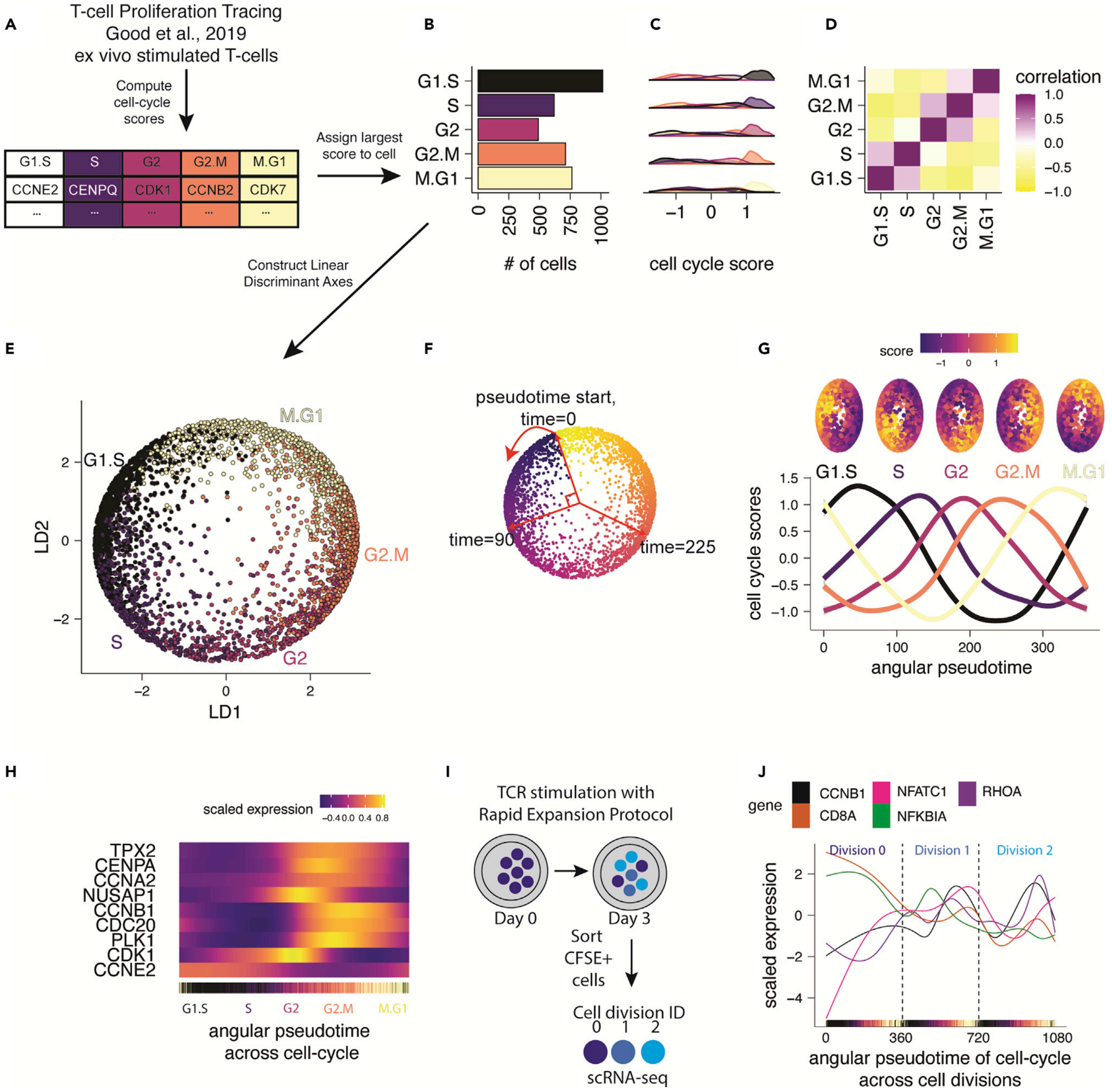

Figure 6. LDA can reconstruct cyclical trajectories using scRNA-seq data

(A) Conceptual diagram of cell-cycle score computation using prior methods on *ex vivo* CD8 T cell TCR stimulation sc-RNA-seq data.
(B) Bar plot summary counts of assigned cell-cycle phase identities. The phase with the largest cell-cycle score is assigned to each cell.
(C) Density plot summary of cell-cycle phases showing enrichment of cell-cycle scores in each respective assigned phase.
(D) Pearson correlation of cell-cycle scores computed across all cells.
(E) Cyclical LDA visualization on cell-cycle scores.
(F) Graphical representation of angular pseudotime calculation.
(G) Generalized linear models of cell-cycle scores across the angular pseudotime estimated using the LD biaxial.
(H) Heatmap of estimated transcript expression summarized as a generalized additive model for key cell-cycle markers across the cell-cycle angular pseudotime.
(I) Conceptual diagram for (J) demonstrating experimental protocol using CFSE-sorted T cells on day 3 of TCR stimulation to extract cell division IDs before 10x Genomics scRNA-seq.
(J) Estimated transcript expression of CyclinB1 and relevant TCR signaling genes identified using derivative analysis plotted across the cell cycle angular pseudotime deconvolved across cell divisions using CFSE-sorted division IDs.

S7B). Given the nature of the continuous cell-cycle trajectory being discretized for prediction on cell-cycle scores, we speculated that cells predicted in an adjacent cell-cycle label may not be incorrect, but instead, likely transitioning. If these cells are removed or counted as correct due to this ambiguity, then the model achieves an accuracy of 99.9%. Classification performance demonstrated that the continuous nature of the cell cycle is captured by LDA, as misclassified cells denote points of transition between cell-cycle phases.

To dissect the circular topology of the cell-cycle LD embedding, we tested the hypothesis that the circularity of the model is dependent on the correlations of the input features. We introduced an equal weight of noise into the cell-cycle scores to reduce the strength of the correlations (Figures S7C and S7D). Equal reduction in the strength of the correlations did not significantly affect the circular embedding, indicating that weak correlations can sufficiently drive a circular trajectory in LDA if the correlative relationships between adjacent class labels are preserved. While balanced disruption of cell-cycle scores to produce weak correlations sufficiently retained a circular embedding, the angular pseudotime estimates fell apart when a significant amount of noise was inserted into the data (Figure S7E). However, introducing noise into three of the cell-cycle scores disrupted the circular, donut shape of the cell-cycle model (Figures S7F–S7H). The imbalanced disruption of cell-cycle scores indicated that the correlative relationships of the input features and their neighbor classes are important for generating models of circular trajectories in a linear transformation technique.

To identify patterns in gene expression associated with the continuous process of the cell cycle, we computed the angular pseudotime from a computationally derived start point (see methods), and projected the cyclical trajectory into a linear temporal space (Figure 6F). To verify that the angular pseudotime represented cell-cycle patterns, we plotted the cell-cycle scores for each phase along the angular pseudotime and found that they increased and decreased according to their expected transitions (Figure 6G). We then observed the expression of key cell-cycle markers over angular pseudotime. We found that the pseudotime expression of CCNB1, CDK1, TPX2, and other cell-cycle genes tracked with prior experimentally proven cell-cycle transitions (Figure 6H). For example, CCNB1 transcription increased during S phase and peaked near the G2/M transition.[27] TPX2, a microtubule-associated protein responsible for microtubule nucleation that is integral for mitosis, also reached peak expression entering mitosis.[28]

To further leverage prior knowledge of experimental conditions with LDA, we used the division ID labels of these CFSE^{+} sorted cells to deconvolve the cell-cycle angular pseudotime across the first two divisions (Figure 6I). We added 0, 360, or 720 to the angular pseudotime value of each cell corresponding to the cell's respective 0, 1, or 2 division ID, resulting in a continuous axis of cell-cycle pseudotime that accounts for the number of divisions a cell has undergone. We observed the gene expression patterning of activated T cells across these divisions, highlighting the expression of relevant TCR signaling genes such as NFKB and NFATC, as well as cell-cycle genes (Figure 6J). Interestingly, NFATC1 expression increased before the first division and began to fluctuate in a cell-cycle-dependent manner. NFKB1A expression decreased before the first division, briefly increased in G1/S of the first division, then began to decrease again. Consistent with our aggregate analysis of cell-cycle gene expression patterns, CCNB1 expression was low in non-proliferating cells and increased with TCR stimulation, eventually following the same strict cell-cycle-dependent pattern in both division 1 and division 2 as cells were proliferating. This serves as an illustration that LDA visualization can be used to derive biologically relevant information beyond initial class labels. This method is robust to noise in cell-cycle scores, and computation of cell-cycle LD axes and angular pseudotime is computationally efficient. Traditional analysis of scRNA-seq data removes cell-cycle effects, blinding analysis to cell-cycle effects. However, the cell cycle is implicated in mediating important biological functions such as cellular differentiation, plasticity, and inflammatory response.[23,29,30] Deconvolving the continuous trajectory of the cell cycle in scRNA-seq data empowers researchers to explore highly resolved cell-cycle-related biological trends. In summary, we found that LDA can visualize cyclical trajectories in scRNA-seq data and that experimentally derived metadata can be leveraged to further deconvolve single-cell patterns across cell cycle and cell division.

DISCUSSION

Here, we applied supervised dimensionality reduction by LDA to visualize and explore a range of single-cell datasets generated by mass cytometry, RNA-seq, ATAC-seq, and CITE-seq. We implemented HSS to identify combinations of features that optimally separate *a priori* classes, providing biologically interpretable axes suitable for visualization as well as other downstream analyses. We benchmarked the performance of LDA against UMAP, PCA, and PHATE and found it often, but not always, outperformed other algorithms across datasets and cell counts.

While most dimensionality-reduction algorithms are unsupervised, HSS-LDA is a supervised method that identifies and highlights differences between designated cell groups. This supervised approach is inappropriate in situations in which labels are unknown, or in which an unbiased view of the data is preferable. LDA, by definition, maximizes class separability and minimizes variance within each projected class, and by doing so will come at the expense of local data structure. However, as many single-cell datasets contain one or more known biological labels, HSS-LDA has wide applicability. Even within a single dataset, the same cells can be visualized across multiple HSS-LDA plots, each highlighting the cellular heterogeneity relevant to a distinct biological label. More generally, supervised computational techniques, including but not limited to LDA, can benefit from information related to experimental design and sample associated metadata. Experiments could be devised such that this metadata could specifically be leveraged by supervised methods during analysis to derive new biological insights.

Other researchers have previously demonstrated the utility of feature selection within a supervised classification framework to study sparse signals in genomic datasets, and HSS is one of many feature selection approaches amenable to discriminant analysis.[16,31,32] Like other feature selection approaches, relevant colinear features may not all be selected by HSS as the separability of the model may not be improved by keeping multiple colinear features. An additional correlation analysis may be

helpful to identify if this issue arises. In addition, HSS enriches for features useful for separating multiple classes and therefore may disregard features only useful for separating a single class. Features selected by HSS are differentially expressed by one or more labeled classes, and follow-up analysis should be performed to understand the nature and statistical significance of these differences. We find that HSS complements LDA as an interpretable feature selection method rendering improved visualizations, but in situations in which HSS is not feasible (>100 dimensions) or desirable, LDA and sda still provide useful plots.

As a linear method, LDA is deterministic, reproducing the same plot from the same cells every time. New, unlabeled data can be visualized on existing LD axes using computationally inexpensive matrix math. LDA does not capture non-linear relationships, which may limit its performance on some datasets. Our findings, however, reinforce the notion that simple, linear models often perform well, even in the presence of non-linear data. While non-linear methods are indispensable in computational biology, linear methods can perform comparably to more complex machine learning approaches, as others have noted.[33] Furthermore, as LD axes are linear combinations of expression or accessibility data, LDA delivers the added benefit of yielding biologically interpretable single-cell coordinates. The linear nature of LDA also facilitates simple out-of-sample extension, in which LD axes are trained on one dataset and then new data are projected onto those axes with computationally efficient matrix math. Here, we demonstrated that utility on the Morphometry dataset, in which diseased samples were visualized on LD axes trained on healthy samples, and on the T cell metabolic regulome dataset, in which the stunted metabolic states of $CD57^{high}$ T cells were visualized on LD axes trained on $CD57^{low}$ T cells. To our knowledge, no other supervised dimensionality-reduction methods provide this functionality.

PCA, the most commonly used linear dimensionality-reduction method, was unsuitable for the visualization of some single-cell data. The perceived shortcomings of linear dimensionality reduction for visualization may, however, primarily reflect only the specific shortcomings of PCA. Indeed, PCA suffers from the crowding problem, in which cell coordinates often overlap in two-dimensional space. While LDA can also manifest some degree of crowding, the problem is largely avoided as axes are specifically generated to separate cell groups of interest from one another. UMAP's solution to crowding is to require a minimum distance between the coordinates of any two cells, which is effective in most situations, but was problematic in our imbalanced datasets. While PCA is not always appropriate for 2D visualization, the algorithm remains a pillar of high-dimensional data analysis. PCA is widely applied in the preprocessing of single-cell data, and principal components often serve as inputs to non-linear dimensionality-reduction methods like UMAP.[34] Like-wise, we found that inputting HSS-LDs into UMAP improved performance over either algorithm alone in the challenging task of visualizing a multi-label, single-cell dataset. LD axes may therefore have added value in processing and analysis of single-cell data, outside of only 2D visualization. This is opposed to non-linear dimensionality reduction that is not typically used beyond single-cell data embedding.

We recommend HSS-LDA to be used on any single-cell datasets in which *a priori* labels are known and expected to segregate cells into somewhat homogeneous groups. This includes labeling cell types, cell states, stimulation time points, and spatially distinct cell subsets. LDA is computationally inexpensive and requires no tuning parameters, so it can be deployed with minimal time investment. This is particularly important for researchers with limited access to high-performance computing resources. LDA can also be applied to visualize biological units other than single cells to tease out differences in summary statistics between samples.[35,36] HSS-LDA may not perform well in situations in which a large degree of heterogeneity exists within a given class. For example, labeling PBMCs according to the time point of origin would likely fail to produce a visualization that segregates time points, as each PBMC sample would be composed of many cell types. Instead, comparing individual cell types (e.g., monocytes) between time points would be more fruitful. In addition, while no hard limit exists for the maximum number of distinct cell populations that can be visualized by a single HSS-LDA plot, performance will suffer with an increasing number of populations. This maximum threshold will vary by dataset, but at a minimum, three populations are required for a 2D visualization, as LDA generates [# of classes −1] LDs.

We emphasize that HSS-LDA should not replace other dimensionality-reduction methods. We encourage researchers to also apply PCA, UMAP, PHATE, and other algorithms to their datasets to benefit from the unique strengths of each algorithm. Given the diversity of technology used and biology explored by single-cell methods, no dimensionality-reduction algorithm is suitable for every situation. Furthermore, information is always lost in the reduction of high-dimensional data to two dimensions. Visualization is only one aspect of single-cell analysis and should always be supplemented with robust, quantitative, high-dimensional analyses. Supervised dimensionality reduction by HSS-LDA uniquely facilitates interpretable visualization, feature selection, and other downstream analysis utility. We envision HSS-LDA as one of many tools that enable computational biologists to visualize and explore single-cell data.

EXPERIMENTAL PROCEDURES

Resource availability

Lead contact

Requests for information and resources used in this article should be addressed to Dr. Sean C. Bendall (bendall@stanford.edu).

Materials availability

There were no new physical or biological materials generated with this study.

Data and code availability

This paper analyzes publicly available data that are accessible from their respective citations or data repositories outlined in this statement. The endoderm differentiation dataset is derived from a manuscript in preparation, and the minimum data used are available in the "manuscript" folder of the published HSS-LDA github repo. The "Morphometry" data are available as five links in FlowRepository: https://flowrepository.org/ searchable with keyword "morphometry." The "T cell metabolic regulome" repository is Zenodo: https://doi.org/10.5281/zenodo.3951613. The "Chromotyping" manuscript is under preparation, and the corresponding data used in this paper have been deposited at FlowRepository: https://flowrepository.org/id/FR-FCM-Z5EA. The scATACseq data reported in this paper are available under GEO: GSE129785. The enterocyte RNAseq data reported in this paper are available under GEO: GSE109413. The CITEseq data reported in this paper are available under GEO: GSE164378. The "T-cell Proliferation Tracing" data reported in this paper are available under GEO: GSE119139. Original code for HSS-LDA is available with installation instructions in R at https://github.com/mamouzgar/hsslda, archived under https://doi.org/10.5281/zenodo.6555102. Parameters associated with HSS-LDA are described in the figure

CellPress
OPEN ACCESS

legends. Software and parameters used for running all other algorithms are available in Table S1. Any additional information required to reanalyze the data reported in this article is available from the lead contact upon request.

Methods

Dataset transformation and preprocessing

We used only previously published datasets and cell annotations provided by the authors. The CyTOF datasets we used are described in the results and paired with graphical representations. For the Morphometry dataset, we used all cells from a single, healthy donor. For the T cell metabolic regulome dataset, we used healthy CD8 naive T cells from the same donor and sampled an equal number of cells for each time point. For the Chromotyping dataset, we used all cells across all cell lines. The same cells are used as inputs into each algorithm when making any algorithm comparison. CyTOF datasets were transformed using an arcsinh scale of 5 and percentile normalized using a quantile value of 0.999.

The scRNA-seq datasets we used are described in the results and paired with graphical representations. scRNA-seq count tables for both the Enterocyte Differentiation and T cell Proliferation Tracing datasets were extracted from their respective publications. Raw count matrices and corresponding metadata were input in Seurat for downstream preprocessing using the NormalizeData, ScaleData, and RunPCA functions. Data were z scaled before PCA transformation, and PC feature matrices were input into LDA or UMAP. The scATAC-seq matrix was similarly processed in Seurat using the Signac extension. Latent semantic indexing (LSI) reduction was performed before input into LDA or UMAP. CITE-seq data was processed in Seurat using their published multimodal vignette.

HSS

The HSS-LDA algorithm uses a stepwise feature selection approach, calculating a separation score for each feature subset, and selecting a final set of features that best separates classes for visualization. The calculated separation score for assessing class separation implements commonly used metrics such as Euclidean distance or silhouette score, as well as pixel-based metrics we have introduced such as pixel density or PCE scores. Users can also define their own separation metric function for use with HSS. We describe the major steps of HSS-LDA below:

1. *Initialize the feature set:* Perform LDA for all pairwise combinations of features and evaluate the separation score. Select the pair of features with the best score as the initial feature set.
2. *Perform forward stepwise selection:* Add each feature to the current feature subset, perform LDA, and evaluate the separation score. Add the feature that results in the best separation score.
3. *Perform reverse stepwise selection:* Subtract each feature in the current feature set, perform LDA, and evaluate the score. Remove the feature from the feature set that most improves the score. If no feature removal improves the score, then proceed without removing any features.
4. Repeat steps 2–3 until the feature set contains all of the features.
5. *Compile the best scores across feature set sizes:* Compile scores for all of the feature sets evaluated in steps 1–4. For each feature set size (2, 3, … k-1, k features), identify the feature set with the best score.
6. *Compute elbow point and select final model:* Calculate the elbow point of scores against feature size in the list compiled in step 5 to select the final feature set. The elbow point is defined as the data point that is the farthest distance from the straight line connecting the first and last data point on a biaxial dot plot of number of markers (x axis) versus score (y axis). Perform LDA using that feature set to generate the final model.

The *hsslda* R package includes a vignette entilted "hsslda-intro" to guide users on how to use HSS-LDA for dimensionality reduction, feature selection, visualization, and exploratory analysis.

Dimensionality-reduction algorithms

We used six dimensionality-reduction algorithms: LDA, sda, PCA, UMAP, and PHATE plus our HSS-LDA approach. The software versions, accession links, and parameters used are listed in Table S1.

Runtime analysis

Runtimes were measured using base R Sys.time() function immediately before and after each algorithm function call to fairly evaluate all of the algorithm runtimes using the exact same input matrices.

Data subsetting for quantitative benchmarking

All of the algorithms were evaluated using the same input cells for each dataset. For subsampling, cells are randomly sampled without replacement, and each subsample was drawn three times for replicate analysis of runtimes. However, a minimum of 20 cells are sampled across each class in a label to preserve the presence of rare populations in datasets with severe class imbalances (e.g., blast cells in Morphometry or mitotic cells in Chromotyping).

Metrics for evaluating separability of cell populations and performing feature selection

Euclidean distance is the distance between the means of each class label and was computed using the stats:dist() R function. It calculates the pairwise Euclidean distance of all class means and selects the minimum as the score, only rewarding manifolds that separate all labels. Silhouette score was used to determine class label separability using the silhouette coefficient equation and was computed using the cluster:silhouette() R function.[37] The closer the silhouette score is to 1, the better the cluster separability. The closer the silhouette score is to -1, the worse the cluster separability.

PCE score is a measure of class label distribution in a biaxial grid. Biaxial plots were pixelated into a 100 × 100 pixel grid, the entropy of classes in each pixel was evaluated, and the PCE score was the average entropy value of each pixel. Here, we defined PCE as $1 - $ [(entropy)/log2(# of unique class labels)]. The closer the PCE score was to 1, the greater the class separation. Pixel density uses the same pixel grid approach as PCE, and the percentage of each class label in each pixel was evaluated. The lower the average pixel density score, the better the class label separation. PCE score and pixel density were encoded in the hsslda github page.

Calculating cell-cycle pseudotime start point and angular pseudotime

The pseudotime start point of the cell cycle is found by selecting the cell with the largest average G1.S and M.G1 cell-cycle score. The angular pseudotime is calculated by assigning a pseudotime value between 1 and 360 to each cell based on its angular location in the cell-cycle model of the LDA. The angular pseudotime of all cells are then adjusted based on the location of the cell at the pseudotime start point.

UMAP initialization with HSS-LDA

Initialization of UMAP with HSS-linear discriminants can be accomplished in R by inputting the first two HSS-LDs or LDs as a matrix into the "init" variable of the uwot:umap() function.

SUPPLEMENTAL INFORMATION

Supplemental information can be found online at https://doi.org/10.1016/j.patter.2022.100536.

ACKNOWLEDGMENTS

M.A. is supported by the Stanford Immunology training grant T32 AI007290_37. D.R.G. is an awardee of the Bio-X Stanford Interdisciplinary Graduate Fellowship. I.A. is an awardee of the Weizmann Institute of Science-Israel National Postdoctoral Award Program for Advancing Women in Science. S.C.K. is supported by the NIH/NIGMS Cell and Molecular Biology Training Grant (T32GM007276). R.B. is supported by the Stanford Cancer Biology Program and funding from the National Science Scholarship (PhD) from the Agency for Science, Technology, and Research (A*STAR). A.G.T. is supported by a Damon Runyon Cancer Research Foundation (DRCRF) (DRG-118-16) and Stanford Department of Pathology Seed Grant. This study was supported by an EMBO Long-Term Fellowship ALTF 1141–2017 (to F.J.H.), the Novartis Foundation for Medical-Biological Research 16C148 (to F.J.H.), and the Swiss National Science Foundation SNF Early Postdoc Mobility P2ZHP3-171741 (to F.J.H.). In addition, we received support from National Institutes of Health 1DP2OD022550-01 (to S.C.B.), 1R01AG056287-01 (to S.C.B.), 1R01AG057915-01 (to S.C.B.), and 1U24CA224309-01 (to S.C.B.). Conceptual graphics created with BioRender.com.

AUTHOR CONTRIBUTIONS

Conceptualization, D.R.G., M.A., and S.C.B.; methodology, D.R.G. and M.A.; software, M.A. and D.R.G.; formal analysis, M.A.; resources, R.B., I.A., S.C.K.,

A.G.T., F.J.H., D.R.G., M.A., and S.C.B.; data curation, M.A. and D.R.G.; writing, M.A. and D.R.G.; editing, S.C.B., D.R.G., M.A., R.B., and F.J.H.; project supervision, S.C.B.; funding acquisition, S.C.B.

DECLARATION OF INTERESTS

The authors declare no competing interests.

INCLUSION AND DIVERSITY

One or more of the authors of this paper self-identifies as living with a disability. One or more of the authors of this paper self-identifies as a member of the LGBTQ+ community.

Received: February 3, 2022
Revised: March 1, 2022
Accepted: June 3, 2022
Published: June 24, 2022

REFERENCES

1. Tang, F., Barbacioru, C., Wang, Y., Nordman, E., Lee, C., Xu, N., Wang, X., Bodeau, J., Tuch, B.B., Siddiqui, A., et al. (2009). mRNA-Seq whole-transcriptome analysis of a single cell. Nat. Methods *6*, 377–382. http://www.nature.com/articles/nmeth.13.
2. Buenrostro, J.D., Wu, B., Litzenburger, U.M., Ruff, D., Gonzales, M.L., Snyder, M.P., Chang, H.Y., and Greenleaf, W.J. (2015). Single-cell chromatin accessibility reveals principles of regulatory variation. Nature *523*, 486–490. http://www.nature.com/articles/nature14590.
3. Han, A., Glanville, J., Hansmann, L., and Davis, M.M. (2014). Linking T-cell receptor sequence to functional phenotype at the single-cell level. Nat. Biotechnol. *32*, 684–692. http://www.nature.com/articles/nbt.2938.
4. Newell, E.W., Sigal, N., Bendall, S.C., Nolan, G.P., and Davis, M.M. (2012). Cytometry by time-of-flight shows combinatorial cytokine expression and virus-specific cell niches within a continuum of CD8+ T cell phenotypes. Immunity *36*, 142–152. https://linkinghub.elsevier.com/retrieve/pii/S1074761312000040.
5. Stoeckius, M., Hafemeister, C., Stephenson, W., Houck-Loomis, B., Chattopadhyay, P.K., Swerdlow, H., Satija, R., and Smibert, P. (2017). Simultaneous epitope and transcriptome measurement in single cells. Nat. Methods *14*, 865–868. http://www.nature.com/articles/nmeth.4380.
6. Swanson, E., Lord, C., Reading, J., Heubeck, A.T., Genge, P.C., Thomson, Z., Weiss, M.D., Li, X., Savage, A.K., Green, R.R., et al. (2021). Simultaneous trimodal single-cell measurement of transcripts, epitopes, and chromatin accessibility using TEA-seq. Elife *10*, e63632. https://doi.org/10.7554/elife.63632. https://elifesciences.org/articles/63632.
7. Glass. (2020). An Integrated Multi-omic Single-Cell Atlas of Human B Cell Identity. Immunity *53*, 217–232. https://doi.org/10.1016/j.immuni.2020.06.013.
8. Bendall, S.C., Simonds, E.F., Qiu, P., Amir, E.A.D., Krutzik, P.O., Finck, R., Bruggner, R.V., Melamed, R., Trejo, A., Ornatsky, O.I., et al. (2011). Single-cell mass cytometry of differential immune and drug responses across a human hematopoietic continuum. Science *332*, 687–696. https://www.science.org/doi/10.1126/science.1198704.
9. King, H.W., Orban, N., Riches, J.C., Clear, A.J., Warnes, G., Teichmann, S.A., and James, L.K. (2021). Single-cell analysis of human B cell maturation predicts how antibody class switching shapes selection dynamics. Sci. Immunol. *6*, eabe6291. https://www.science.org/doi/10.1126/sciimmunol.abe6291.
10. Pearson, K. (1901). LIII. On lines and planes of closest fit to systems of points in space. Lond. Edinb. Dublin Philos. Mag. J. Sci. *2*, 559–572. https://www.tandfonline.com/doi/full/10.1080/14786440109462720.
11. Becht, E., McInnes, L., Healy, J., Dutertre, C.A., Kwok, I.W.H., Ng, L.G., Ginhoux, F., and Newell, E.W. (2019). Dimensionality reduction for visualizing single-cell data using UMAP. Nat. Biotechnol. *37*, 38–44. http://www.nature.com/articles/nbt.4314.
12. Moon, K.R., van Dijk, D., Wang, Z., Gigante, S., Burkhardt, D.B., Chen, W.S., Yim, K., Elzen, A., Hirn, M.J., Coifman, R.R., et al. (2019). Visualizing structure and transitions in high-dimensional biological data. Nat. Biotechnol. *37*, 1482–1492. https://www.nature.com/articles/s41587-019-0336-3.
13. Tsai, A.G., Glass, D.R., Juntilla, M., Hartmann, F.J., Oak, J.S., Fernandez-Pol, S., Ohgami, R.S., and Bendall, S.C. (2020). Multiplexed single-cell morphometry for hematopathology diagnostics. Nat. Med. *26*, 408–417. http://www.nature.com/articles/s41591-020-0783-x.
14. Hastie, T., Tibshirani, R., and Friedman, J.H. (2009). The Elements of Statistical Learning: Data Mining, Inference, and Prediction, Second Edition (Springer).
15. Hartmann, F.J., Mrdjen, D., McCaffrey, E., Glass, D.R., Greenwald, N.F., Bharadwaj, A., Khair, Z., Verberk, S.G.S., Baranski, A., Baskar, R., et al. (2021). Single-cell metabolic profiling of human cytotoxic T cells. Nat. Biotechnol. *39*, 186–197. https://www.nature.com/articles/s41587-020-0651-8.
16. Clemmensen, L., Vernal, D.L., and Steinhausen, H.C. (2012). A systematic review of the long-term outcome of early onset schizophrenia. BMC Psychiatr. *12*, 150. https://doi.org/10.1186/1471-244x-12-150. https://bmcpsychiatry.biomedcentral.com/articles/10.1186/1471-244X-12-150.
17. Kimmey, S.C., Borges, L., Baskar, R., and Bendall, S.C. (2019). Parallel analysis of tri-molecular biosynthesis with cell identity and function in single cells. Nat. Commun. *10*, 1185. https://doi.org/10.1038/s41467-019-09128-7. http://www.nature.com/articles/s41467-019-09128-7.
18. Bartkova, J., Lukas, J., and Bartek, J. (1997). Aberrations of the G1- and G1/S-regulating genes in human cancer. In Progress in Cell Cycle Research, L. Meijer, S. Guidet, and M. Philippe, eds. (Springer US), pp. 211–220. http://link.springer.com/10.1007/978-1-4615-5371-7_16.
19. Kobak, D., and Linderman, G.C. (2021). Initialization is critical for preserving global data structure in both t-SNE and UMAP. Nat. Biotechnol. *39*, 156–157. http://www.nature.com/articles/s41587-020-00809-z.
20. Moor, A.E., Harnik, Y., Ben-Moshe, S., Massasa, E.E., Rozenberg, M., Eilam, R., Bahar Halpern, K., and Itzkovitz, S. (2018). Spatial reconstruction of single enterocytes uncovers broad zonation along the intestinal villus axis. Cell *175*, 1156–1167.e15. https://linkinghub.elsevier.com/retrieve/pii/S0092867418311644.
21. Satpathy, A.T., Granja, J.M., Yost, K.E., Qi, Y., Meschi, F., McDermott, G.P., Olsen, B.N., Mumbach, M.R., Pierce, S.E., Corces, M.R., et al. (2019). Massively parallel single-cell chromatin landscapes of human immune cell development and intratumoral T cell exhaustion. Nat. Biotechnol. *37*, 925–936. http://www.nature.com/articles/s41587-019-0206-z.
22. Hao, Y., Hao, S., Andersen-Nissen, E., Mauck, W.M., Zheng, S., Butler, A., Lee, M.J., Wilk, A.J., Darby, C., Zager, M., et al. (2021). Integrated analysis of multimodal single-cell data. Cell *184*, 3573–3587.e29. https://linkinghub.elsevier.com/retrieve/pii/S0092867421005833.
23. Good, Z., Borges, L., Vivanco Gonzalez, N., Sahaf, B., Samusik, N., Tibshirani, R., Nolan, G.P., and Bendall, S.C. (2019). Proliferation tracing with single-cell mass cytometry optimizes generation of stem cell memory-like T cells. Nat. Biotechnol. *37*, 259–266. http://www.nature.com/articles/s41587-019-0033-2.
24. Macosko, E.Z., Basu, A., Satija, R., Nemesh, J., Shekhar, K., Goldman, M., Tirosh, I., Bialas, A.R., Kamitaki, N., Martersteck, E.M., et al. (2015). Highly parallel genome-wide expression profiling of individual cells using nanoliter droplets. Cell *161*, 1202–1214. https://linkinghub.elsevier.com/retrieve/pii/S0092867415005498.
25. Schwabe, D., Formichetti, S., Junker, J.P., Falcke, M., and Rajewsky, N. (2020). The transcriptome dynamics of single cells during the cell cycle. Mol. Syst. Biol. *16*. https://onlinelibrary.wiley.com/doi/10.15252/msb.20209946.
26. Whitfield, M.L., Sherlock, G., Saldanha, A.J., Murray, J.I., Ball, C.A., Alexander, K.E., Matese, J.C., Perou, C.M., Hurt, M.M., Brown, P.O., and Botstein, D. (2002). Identification of genes periodically expressed in

CellPress
OPEN ACCESS

the human cell cycle and their expression in tumors. Mol. Biol. Cell *13*, 1977–2000. https://www.molbiolcell.org/doi/10.1091/mbc.02-02-0030.

27. Farshadi, E., Yan, J., Leclere, P., Goldbeter, A., Chaves, I., and van der Horst, G.T.J. (2019). The positive circadian regulators CLOCK and BMAL1 control G2/M cell cycle transition through cyclin B1. Cell Cycle *18*, 16–33. https://www.tandfonline.com/doi/full/10.1080/15384101.2018.1558638.
28. Stewart, S., and Fang, G. (2005). Anaphase-promoting complex/cyclosome controls the stability of TPX2 during mitotic exit. Mol. Cell Biol. *25*, 10516–10527. https://journals.asm.org/doi/10.1128/MCB.25.23.10516-10527.2005.
29. Li, V.C., and Kirschner, M.W. (2014). Molecular ties between the cell cycle and differentiation in embryonic stem cells. Proc. Natl. Acad. Sci. *111*, 9503–9508. https://pnas.org/doi/full/10.1073/pnas.1408638111.
30. Daniel, B., Belk, J.A., Meier, S.L., Chen, A.Y., Sandor, K., Qi, Y., Kitano, H., Wheeler, J.R., Foster, D.S., Januszyk, M., and Longaker, M.T. (2021). Macrophage inflammatory and regenerative response periodicity is programmed by cell cycle and chromatin state. Immunology. http://biorxiv.org/lookup/doi/10.1101/2021.06.24.449850.
31. Lê Cao, K.A., Boitard, S., and Besse, P. (2011). Sparse PLS discriminant analysis: biologically relevant feature selection and graphical displays for multiclass problems. BMC Bioinf. *12*, 253. https://doi.org/10.1186/1471-2105-12-253. https://bmcbioinformatics.biomedcentral.com/articles/10.1186/1471-2105-12-253.
32. Witten, D.M., and Tibshirani, R. (2010). A framework for feature selection in clustering. J. Am. Stat. Assoc. *105*, 713–726. http://www.tandfonline.com/doi/abs/10.1198/jasa.2010.tm09415.
33. Christodoulou, E., Ma, J., Collins, G.S., Steyerberg, E.W., Verbakel, J.Y., and Van Calster, B. (2019). A systematic review shows no performance benefit of machine learning over logistic regression for clinical prediction models. J. Clin. Epidemiol. *110*, 12–22. https://doi.org/10.1016/j.jclinepi.2019.02.004. https://linkinghub.elsevier.com/retrieve/pii/S0895435618310813.
34. Luecken, M.D., and Theis, F.J. (2019). Current best practices in single-cell RNA-seq analysis: a tutorial. Mol. Syst. Biol. *15*. https://onlinelibrary.wiley.com/doi/10.15252/msb.20188746.
35. Jiang, S., Chan, C.N., Rovira-Clave, X., Chen, H., Bai, Y., Zhu, B., McCaffrey, E., Greenwald, N.F., Liu, C., Barlow, G.L., and Weirather, J.L. (2021). Virus-dependent immune conditioning of tissue microenvironments. Immunology. http://biorxiv.org/lookup/doi/10.1101/2021.05.21.444548.
36. Moore, A.R., Gonzalez, N.V., Plummer, K.A., Mitchel, O.R., Kaur, H., Rivera, M., Collica, B., Palmer, T., and Bendall, S.C. (2021). Gestationally-dependent immune organization at the maternal-fetal interface. Immunology. http://biorxiv.org/lookup/doi/10.1101/2021.06.25.449807.
37. Rousseeuw. (1987). Silhouettes: A graphical aid to the interpretation and validation of cluster analysis. Journal of Computational and Applied Mathematics. https://doi.org/10.1016/0377-0427(87)90125-7.

Patterns, Volume *3*

Supplemental information

Supervised dimensionality reduction for exploration of single-cell data by HSS-LDA

Meelad Amouzgar, David R. Glass, Reema Baskar, Inna Averbukh, Samuel C. Kimmey, Albert G. Tsai, Felix J. Hartmann, and Sean C. Bendall

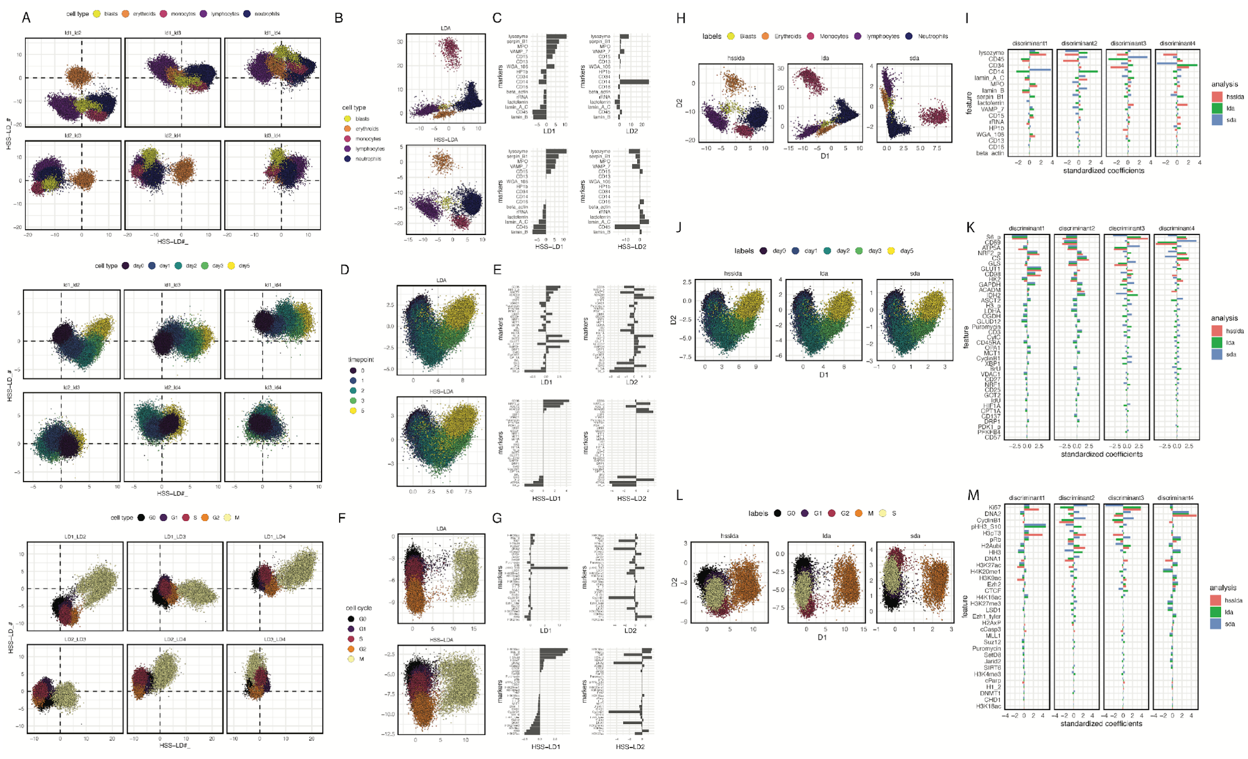

Figure S1: Comparison of LDA to HSS-LDA dimensionality reduction for visualization, feature selection, and feature importance of different mass cytometry datasets.
(A) Pairwise plots of HSS-LDs for the 3 primary mass cytometry datasets. **(B-F)** Results comparing LDA vs HSS-LDA on the same 100,000 subset of cells for each of the 3 primary mass cytometry datasets. **(B, D, F)** LDA and HSS-LDA embeddings colored by cell-type, collection timepoint, and cell cycle phase for the Morphometry, T-cell metabolic regulome, and Chromotyping datasets, respectively. **(C, E, G)** LDA coefficients and HSS-LDA coefficients for the first two linear discriminants sorted by HSS-LDA coefficient values. Features at zero were removed by HSS-LDA. **(H-M)** Comparison of LDA (lda), HSS-LDA (hsslda), and sparseLDA (sda) on the same input cells for each mass cytometry dataset.

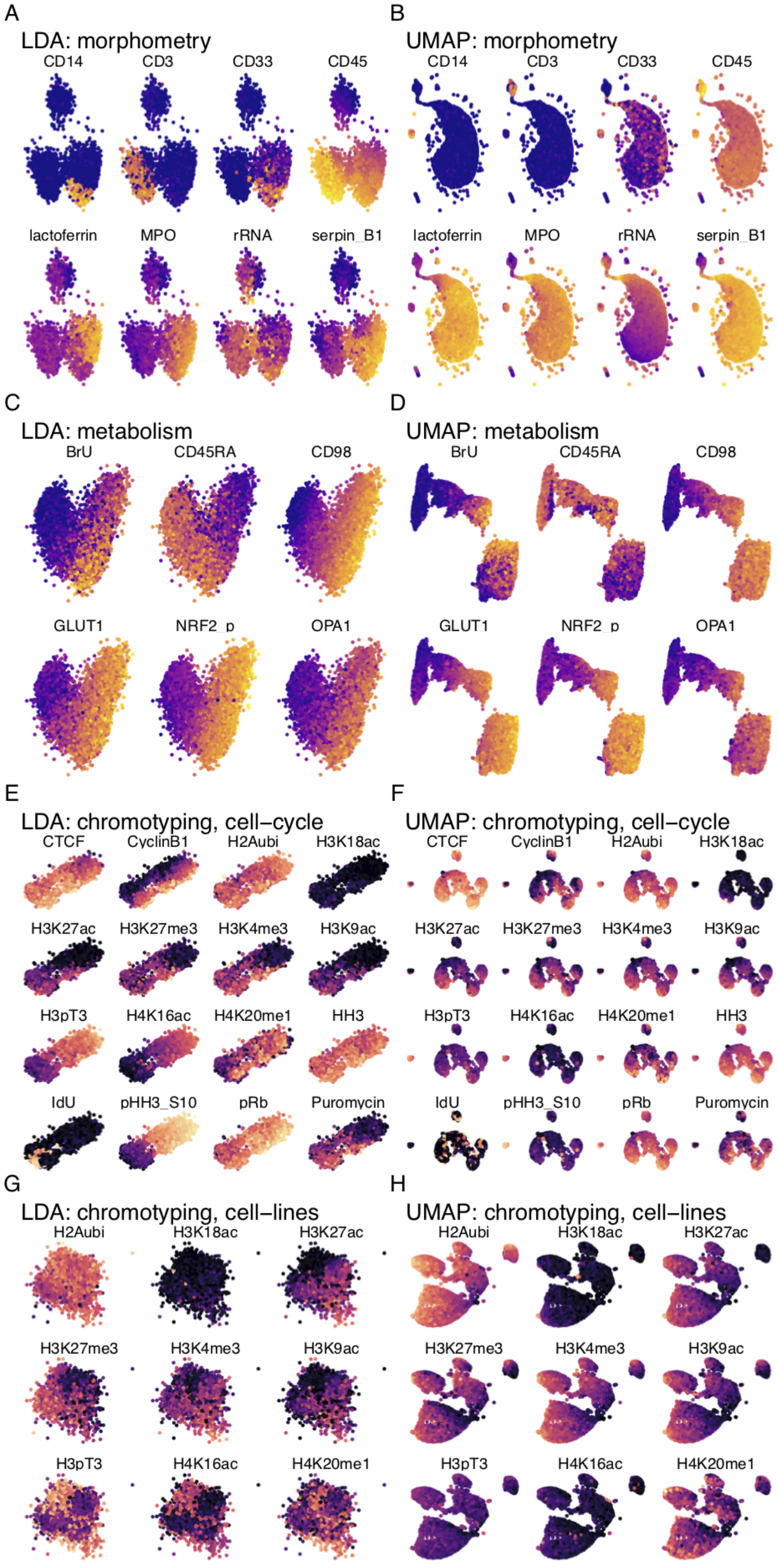
A
LDA: morphometry
CD14
CD3
CD33
CD45
lactoferrin
MPO
rRNA
serpin_B1
B
UMAP: morphometry
CD14
CD3
CD33
CD45
lactoferrin
MPO
rRNA
serpin_B1
C
LDA: metabolism
BrU
CD45RA
CD98
GLUT1
NRF2_p
OPA1
D
UMAP: metabolism
BrU
CD45RA
CD98
GLUT1
NRF2_p
OPA1
E
LDA: chromotyping, cell–cycle
CTCF
CyclinB1
H2Aubi
H3K18ac
H3K27ac
H3K27me3
H3K4me3
H3K9ac
H3pT3
H4K16ac
H4K20me1
HH3
IdU
pHH3_S10
pRb
Puromycin
F
UMAP: chromotyping, cell–cycle
CTCF
CyclinB1
H2Aubi
H3K18ac
H3K27ac
H3K27me3
H3K4me3
H3K9ac
H3pT3
H4K16ac
H4K20me1
HH3
IdU
pHH3_S10
pRb
Puromycin
G
LDA: chromotyping, cell–lines
H2Aubi
H3K18ac
H3K27ac
H3K27me3
H3K4me3
H3K9ac
H3pT3
H4K16ac
H4K20me1
H
UMAP: chromotyping, cell–lines
H2Aubi
H3K18ac
H3K27ac
H3K27me3
H3K4me3
H3K9ac
H3pT3
H4K16ac
H4K20me1

Figure S2: Protein expression of HSS-LDA and UMAP embeddings for different mass cytometry datasets.
(A) Morphometry HSS-LDA embedding colored by protein expression for cell-type labels. **(B)** Morphometry UMAP embedding colored by protein expression for cell-type labels. **(C)** T-cell metabolic regulome HSS-LDA embedding colored by protein expression for for timepoint labels. **(D)** T-cell metabolic regulome UMAP embedding colored by protein expression for timepoint labels. **(E)** Chromotyping HSS-LDA embedding colored by protein expression for cell cycle labels. **(F)** Chromotyping UMAP embedding colored by protein expression for cell cycle labels. **(G)** Chromotyping HSS-LDA embedding colored by protein expression for cell line labels. **(H)** Chromotyping UMAP embedding colored by protein expression for cell line labels.

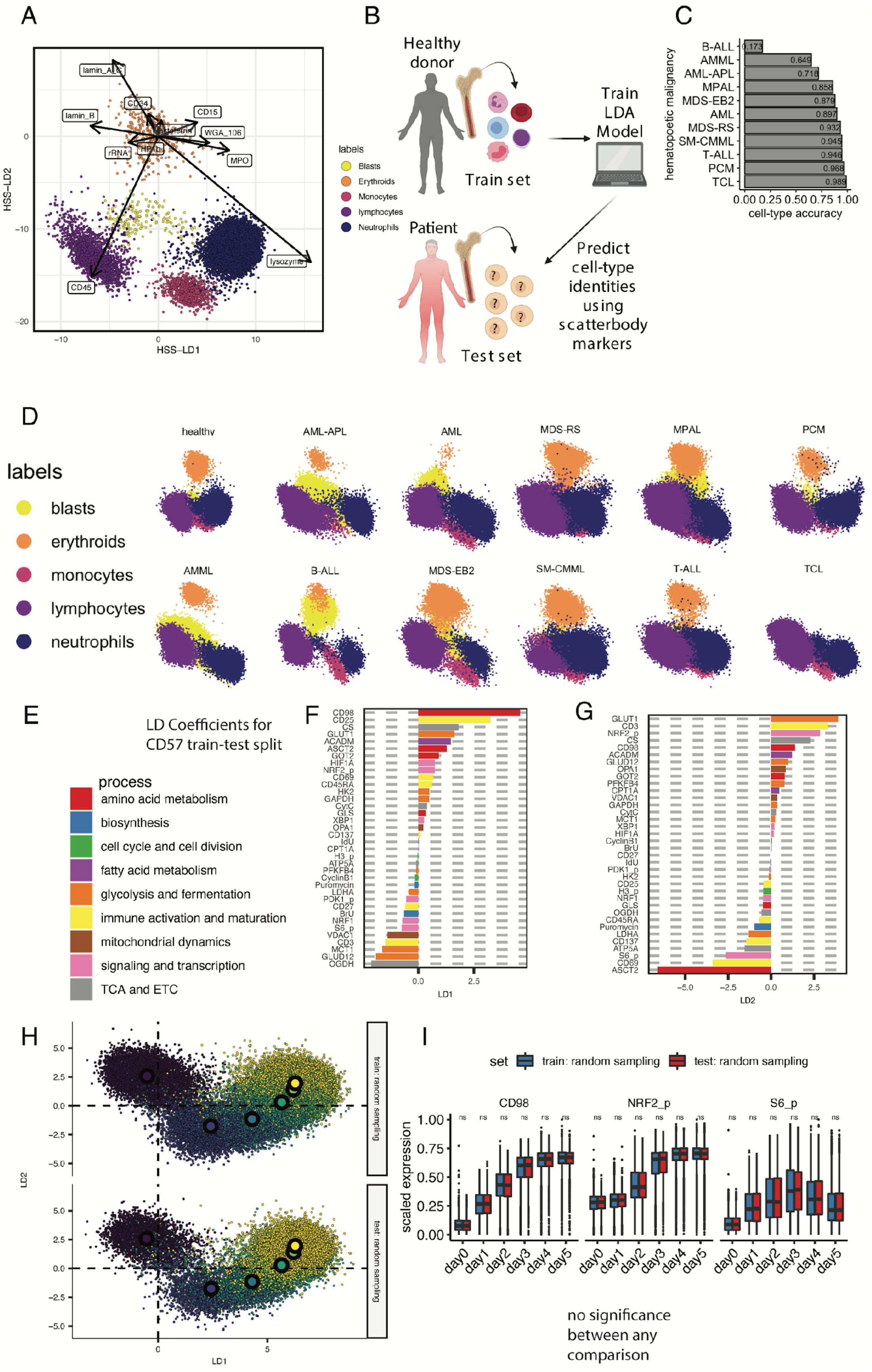
A
B
C
HSS–LD1
HSS–LD2
lamin_A/C
lamin_B
CD34
CD15
WGA_106
rRNA
MPO
lysozyme
CD45
labels
Blasts
Erythroids
Monocytes
lymphocytes
Neutrophils
Healthy donor
Train set
Train LDA Model
Patient
Test set
Predict cell-type identities using scatterbody markers
hematopoetic malignancy
cell-type accuracy
B-ALL 0.173
AMML 0.649
AML-APL 0.718
MPAL 0.858
MDS-EB2 0.879
AML 0.897
MDS-RS 0.932
SM-CMML 0.945
T-ALL 0.946
PCM 0.968
TCL 0.989
0.00 0.25 0.50 0.75 1.00
D
labels
blasts
erythroids
monocytes
lymphocytes
neutrophils
healthy
AML-APL
AML
MDS-RS
MPAL
PCM
AMML
B-ALL
MDS-EB2
SM-CMML
T-ALL
TCL
E
LD Coefficients for CD57 train-test split
process
amino acid metabolism
biosynthesis
cell cycle and cell division
fatty acid metabolism
glycolysis and fermentation
immune activation and maturation
mitochondrial dynamics
signaling and transcription
TCA and ETC
F
LD1
G
LD2
H
LD1
LD2
train: random sampling
test: random sampling
I
set
train: random sampling
test: random sampling
CD98
NRF2_p
S6_p
scaled expression
day0
day1
day2
day3
day4
day5
no significance between any comparison

Figure S3: HSS-LDA for predictive analysis of cell-types across different hematopoietic malignancies using scatterbodies, and random sampling of T-cell metabolism dataset for projecting unseen data.
(A) Biaxial plot of LD coefficients representing the magnitude and direction for each feature selected by HSS-LDA. **(B)** Graphical illustration of training HSS-LDA model on immune cells from healthy patients using scatterbody protein markers and tested on immune cells from patients with diverse hematopoietic malignancies for accurate cell-type predictions. **(C)** Accuracy of cell-type predictions across different hematopoietic malignancies using HSS-LDA. **(D)** Biaxial LD embeddings of training set (healthy) and test sets (hematopoietic malignancies) projected onto the healthy LD embedding space. **(E)** Labels for different biological system categories of each marker, derived from Hartmann et al, 2020. **(F-G)** Magnitude and direction of coefficients for all markers in the HSS-LD1 *(E)* and HSS-LD2 *(F)* axes, respectively. **(H)** Biaxial HSS-LD plots of randomly sampled cells independent of CD57 expression and labeled with the centroid point for each timepoint. Supervised learning models are known to have decreased performance on test sets. To test the hypothesis that the metabolically slowed trajectory of $CD57^{high}$ cells seen in *G-I* is not due to decreased performance on the $CD57^{high}$ test set, we randomly sample cells for a train-test split and train the HSS-LDA model. The biaxial HSS-LD plots show there is no change between the training set and test set, indicating the metabolically slowed progression phenotype observed in $CD57^{high}$ cells is not dependent on poor test set performance. **(I)** Boxplot summary of protein expression for randomly sampled cells across each timepoint. Wilcoxon signed-rank test performed between randomly sampled cells across each timepoint shows no significant difference in protein expression between the train or test set, indicating the metabolically slowed phenotype in the $CD57^{high}$ cells versus $CD57^{low}$ (Figure 2) is a true representation of their metabolic trajectory. Wilcoxon signed-rank test performed between $CD57^{low}$ and $CD57^{high}$ cells across each timepoint. *: $p <= 0.05$; **: $p <= 0.01$; ***: $p <= 0.001$; ****: $p <= 0.0001$.

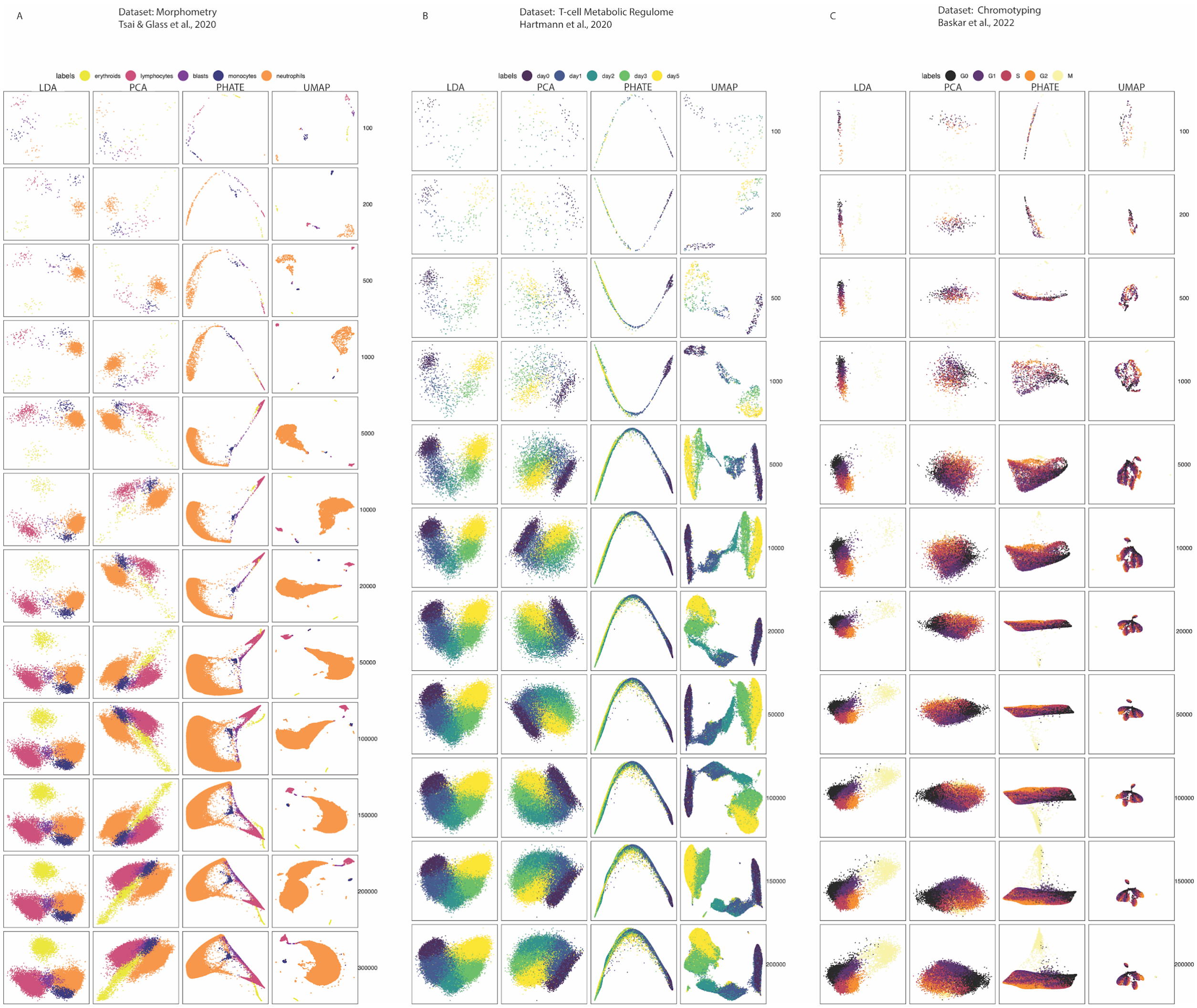

Figure S4: Comparison of biaxial embeddings for LDA, PCA, UMAP, and PHATE across different data subsets.

(A-C) Biaxial visualizations for varying cell-counts across each algorithm using the **(A)** Morphometry for cell-type, **(B)** T-cell Metabolic Regulome for timepoints, and **(C)** Chromotyping for cell cycle datasets. All algorithms benefit from HSS-LDA feature selection, and the feature matrix for each algorithm is the same for each subset of the data.

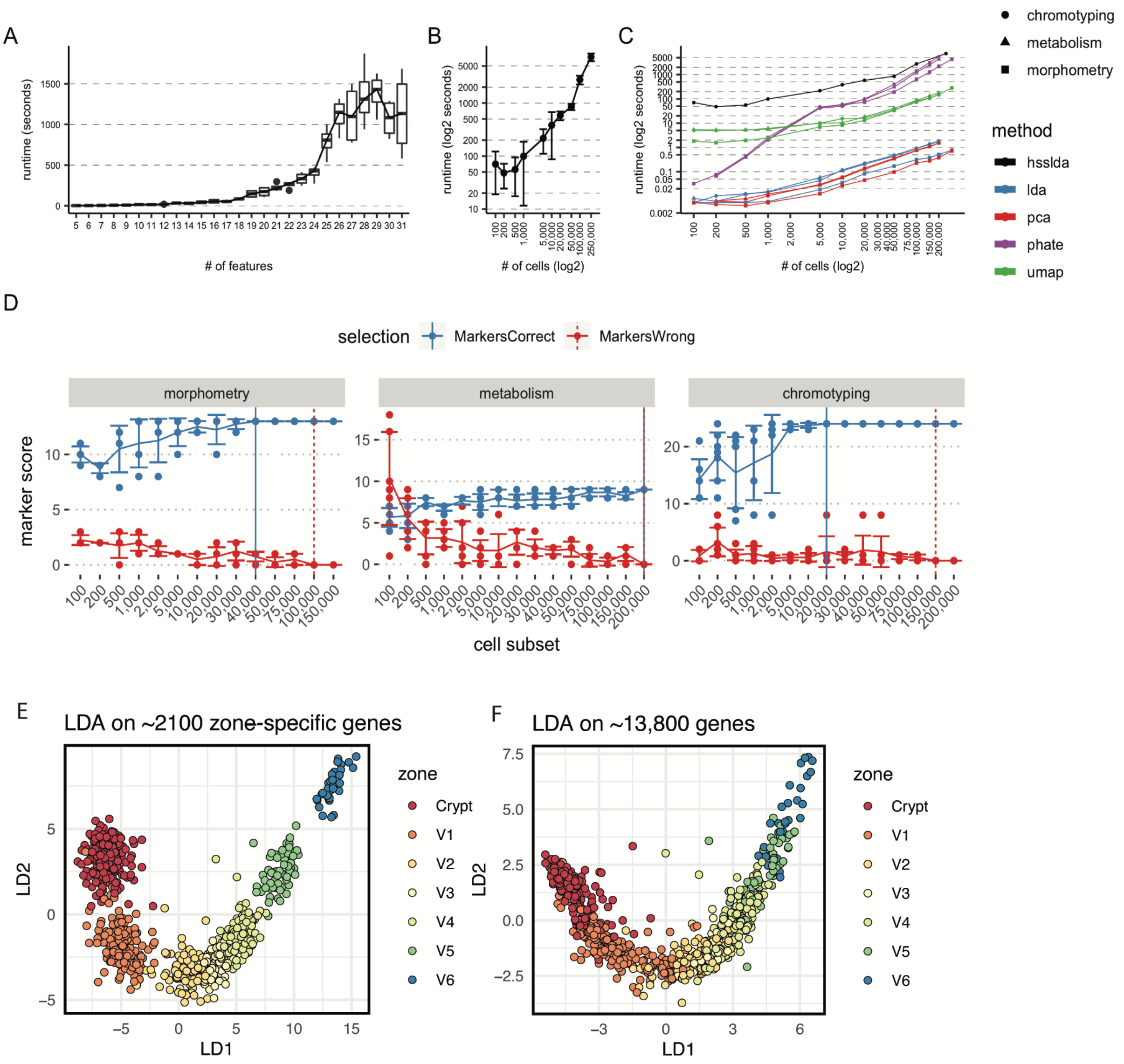

Figure S5: HSS-LDA runtime analysis and assessment of final feature selection.
(A) Runtime analysis for HSS-LDA with varying # of starting markers randomly selected using the same 50,000 cells. **(B)** Runtime analysis with mean and standard deviation for varying # of cells using 32 chromotyping markers as input feature set. **(C)** Runtime analysis of HSS-LDA benchmarked against all other algorithms that do not perform feature selection. **(D)** Assessment of the minimum # of cells required for HSS-LDA to select the feature set that maximally separates class labels. **(E)** LDA directly on the ~2,100 zone-specific genes published by Moor et al., 2018. Runtime was approximately 12.6 seconds. **(F)** LDA directly on the ~13,800 genes after initial preprocessing to remove genes with low variable expression across the dataset. Runtime was approximately ~6.4 minutes.

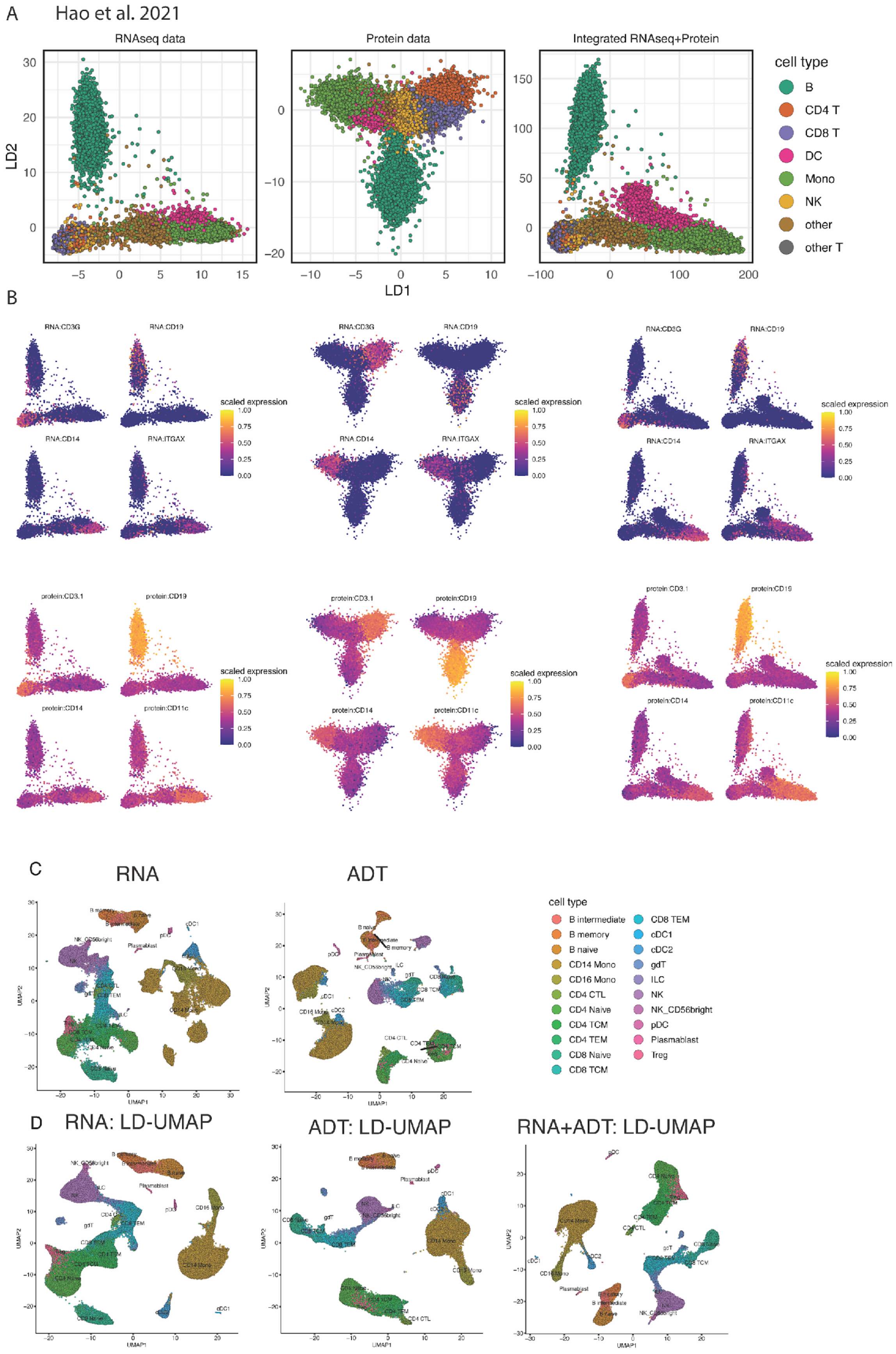
A
Hao et al. 2021
RNAseq data
Protein data
Integrated RNAseq+Protein
LD2
LD1
cell type
B
CD4 T
CD8 T
DC
Mono
NK
other
other T
B
RNA:CD3G
RNA:CD19
RNA:CD14
RNA:ITGAX
protein:CD3.1
protein:CD19
protein:CD14
protein:CD11c
scaled expression
C
RNA
ADT
cell type
B intermediate
B memory
B naive
CD14 Mono
CD16 Mono
CD4 CTL
CD4 Naive
CD4 TCM
CD4 TEM
CD8 Naive
CD8 TCM
CD8 TEM
cDC1
cDC2
gdT
ILC
NK
NK_CD56bright
pDC
Plasmablast
Treg
D
RNA: LD-UMAP
ADT: LD-UMAP
RNA+ADT: LD-UMAP
UMAP1
UMAP2

Figure S6: Supervised dimensionality reduction by LDA for multi-omic data integration
Dimensionality reduction by UMAP or LD-UMAP using multi-omic CITE-seq dataset of 154,491 cells from Hao et al., 2021. **(A)** Biaxial plot of LD1 and LD2 from LDA on PCs 1 to 30 supervised with general immune cell type annotations provided by the original authors for downstream analysis shown with only RNA data *(left)*, only ADT data *(center)*, and both the RNA and protein PCs *(right)*. **(B)** Scaled RNA and protein expression for select gene and protein targets. **(C-D)** Dimensionality reduction by UMAP or LD-UMAP using multi-omic CITE-seq dataset of 154,491 immune cells from Hao et al., 2021. All uwot::umap parameters were the same: n_neighbors = 50, spread = 4, min_dist = 0.1. **(C)** Unsupervised dimensionality reduction by UMAP on RNA (left) and ADT (right) on the first 50 PCs colored by celltype. **(D)** Supervised dimensionality reduction of first 50 PCs by LDA using previously published cell-type annotations determined from RNA or ADT data followed by input of all LDs into UMAP for RNA (left) or ADT (center) data. Multi-omic integration of LDs for RNA and ADT inputted into UMAP (right).

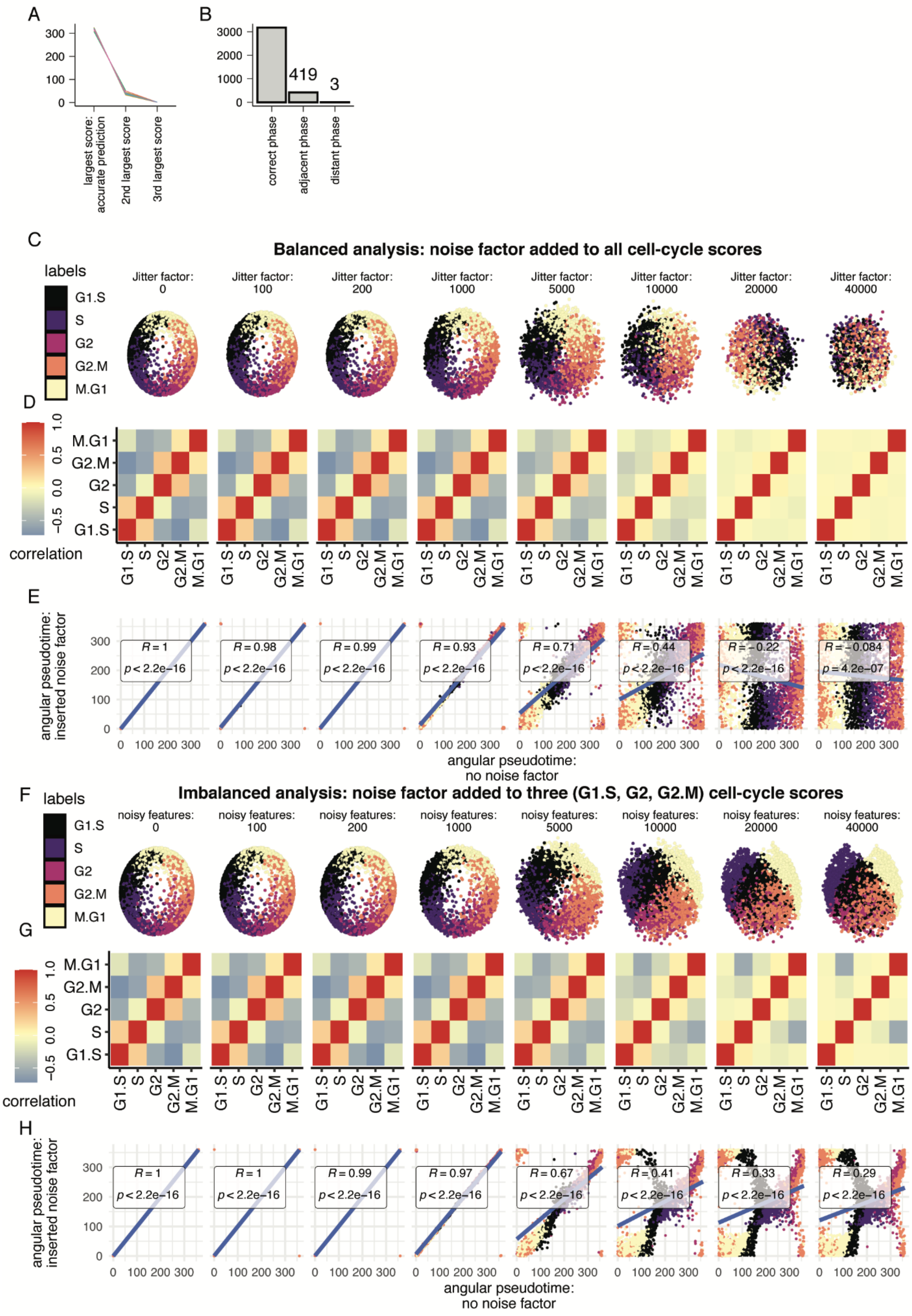
A
B
largest score: accurate prediction
2nd largest score
3rd largest score
correct phase
adjacent phase
distant phase
419
3
C
Balanced analysis: noise factor added to all cell-cycle scores
labels
G1.S
S
G2
G2.M
M.G1
Jitter factor: 0
Jitter factor: 100
Jitter factor: 200
Jitter factor: 1000
Jitter factor: 5000
Jitter factor: 10000
Jitter factor: 20000
Jitter factor: 40000
D
correlation
E
angular pseudotime: inserted noise factor
angular pseudotime: no noise factor
F
Imbalanced analysis: noise factor added to three (G1.S, G2, G2.M) cell-cycle scores
noisy features: 0
noisy features: 100
noisy features: 200
noisy features: 1000
noisy features: 5000
noisy features: 10000
noisy features: 20000
noisy features: 40000
G
H

Figure S7: Cross-validation and noise-insertion analysis shows that cell cycle LDA is an accurate embedding of cell cycle progression and that correlative relationships are essential for constructing circular trajectory models in linear transformation techniques.
(A) Summary barplot of 10-fold cross-validation accuracy results for cyclical LDA model performed on non-overlapping test sets. Accurate cell cycle phase predictions match the largest cell cycle score. 88% test set accuracy. **(B)** Barplot summary of test set predictions indicating cell assignment predictions as either the correct cell cycle phase, an adjacent cell cycle phase, or a distant cell cycle phase. Incorrect predictions are often cells transitioning through the cell cycle in an adjacent cell cycle phase. **(C)** LD embeddings of cell cycle LDAs with increasing amounts of equal noise inserted into all cell cycle scores. **(D)** Correlation heatmaps of cell cycle scores with increasing amounts of equal noise inserted into all cell cycle scores. **(E)** Angular pseudotime estimates of the noisy model versus unperturbed model. A simple linear regression model was generated to determine R2 and P value. **(F)** LD embeddings of cell cycle LDAs with increasing amounts of imbalanced noise inserted into three cell cycle scores (G1.S, G2, G2.M). **(G)** Correlation heatmaps of cell cycle scores with increasing amounts of imbalanced noise inserted into three cell cycle scores. **(H)** Angular pseudotime estimates of the noisy model versus unperturbed model. A simple linear regression model was generated to determine R2 and P value.

Supplementary Table #1:

Algorithm	Version	Availability	parameters
HSS-LDA	0.1	https://github.com/mamouzgar/hsslda	euclidean or PCE score feature selection, specified in figure legends
LDA	MASS: 7.3-51.5	https://cran.r-project.org/web/packages/MASS/index.html	NA
PCA	stats: 3.6.1	Base R package	NA
UMAP	uwot: 0.1.10	https://cran.r-project.org/web/packages/uwot/index.html	default unless otherwise specified in figure legends
PHATE	PHATE: v0.3.0	https://github.com/KrishnaswamyLab/PHATE#r	default unless otherwise specified in figure legends
sparse LDA	sparseLDA: v0.1.9	https://cran.r-project.org/web/packages/sparseLDA/index.html	default unless otherwise specified in figure legends

Table S1: Software versions, accession links, and parameters used for different algorithms.
